# Assignment of Ala, Ile, Leu^proS^, Met, and Val^proS^ methyl groups of the protruding domain of murine norovirus capsid protein VP1 using methyl–methyl NOEs, site directed mutagenesis, and pseudocontact shifts

**DOI:** 10.1007/s12104-022-10066-7

**Published:** 2022-01-20

**Authors:** Thorben Maass, Leon Torben Westermann, Robert Creutznacher, Alvaro Mallagaray, Jasmin Dülfer, Charlotte Uetrecht, Thomas Peters

**Affiliations:** 1 Center of Structural and Cell Biology in Medicine (CSCM), Institute of Chemistry and Metabolomics, University of Luebeck, Ratzeburger Allee 160, 23562 Luebeck, Germany; 2 Leibniz Institute for Experimental Virology (HPI), 20251 Hamburg, Germany; 3 School of Life Sciences, University of Siegen, 57076 Siegen & Centre for Structural Systems Biology (CSSB), & Deutsches Elektronensynchrotron (DESY), 22607 Hamburg & European XFEL GmbH, 22869 Schenefeld, Germany

**Keywords:** Methyl group assignment, Pseudo contact shifts, Norovirus, P-domain, 4D HMQC-NOESY-HMQC, Mass spectrometry, Peptide mapping, Proline *cis*/*trans*

## Abstract

The protruding domain (P-domain) of the murine norovirus (MNV) capsid protein VP1 is essential for infection. It mediates receptor binding and attachment of neutralizing antibodies. Protein NMR studies into interactions of the P-domain with ligands will yield insights not easily available from other biophysical techniques and will extend our understanding of MNV attachment to host cells. Such studies require at least partial NMR assignments. Here, we describe the assignment of about 70% of the Ala, Ile, Leu^proS^, Met, and Val^proS^ methyl groups. An unfavorable distribution of methyl group resonance signals prevents complete assignment based exclusively on 4D HMQC-NOESY-HMQC experiments, yielding assignment of only 55 out of 100 methyl groups. Therefore, we created point mutants and measured pseudo contact shifts, extending and validating assignments based on methyl-methyl NOEs. Of note, the P-domains are present in two different forms caused by an approximate equal distribution of *trans*- and *cis*-configured proline residues in position 361.

**Supplementary Information:**

The online version contains supplementary material available at 10.1007/s12104-022-10066-7.

## Biological context

The genus norovirus belongs to the family of *Caliciviridae*, a family of positive-sense single-stranded RNA viruses. Human noroviruses (HuNoV) are the major cause for acute viral gastroenteritis and constitute a severe burden to health care systems, not only in developing countries (Widdowson et al. 2005; Bányai et al. 2018; Robilotti et al. 2015). According to estimates, HuNoVs are responsible for over 200,000 deaths each year (Lopman et al. 2016). Cell culture systems for HuNoVs became available only recently and are still not trivial to implement (Ettayebi et al. 2016). Therefore, the closely related murine Norovirus (MNV) has been established as a model system (Wobus et al. 2006). A cell-culture system (Wobus et al. 2004), a reverse genetics system (Ward et al. 2007), and a small animal model allow studying biological aspects of MNV infection (Vashist et al. 2009; Taube et al. 2013).

Understanding the molecular mechanisms of MNV host cell entry as a first step of infection requires knowledge about structure and dynamics of the viral capsid. The capsid consists of 180 copies of one protein, VP1, which is subdivided into a shell domain (S-domain) and a protruding domain (P-domain) (Prasad et al. 1999, 1994). The S-domain forms the capsid itself, whereas the P-domain has binding sites for the host-cell receptor, neutralizing antibodies, and attachment factors. Notably, bile acids have been identified as important attachment factors. Bile acids promote infection (Nelson et al. 2018) and induce transition of viral capsids from an extended, "open", conformation into a compressed, "closed", conformation (Williams et al. 2021; Sherman et al. 2019; Creutznacher et al. 2021a). The effect is strongly dependent on bile acid type, with glycochenodeoxycholic acid (GCDCA) being the most effective agent. The "open" state is characterized by a P-domain hovering 10 to 15 Å above the shell (Williams et al. 2021; Sherman et al. 2019), and likely with the P-domains only loosely associated as dimers (P-dimers) (Creutznacher et al. 2021a). In the presence of GCDCA the capsid contracts into the "closed" state, stabilizing the P-dimers and placing them in close proximity to the S-domain (Williams et al. 2021; Sherman et al. 2019). It appears that these complex conformational transitions also depend on bivalent metal ions, promoting infection and influencing P-domain receptor-binding (Orchard et al. 2016; Nelson et al. 2018; Kilic et al. 2018; Graziano et al. 2019, 2021; Williams et al. 2021; Song et al. 2020). Other co-factors may still be unidentified. Even though several crystal structures of P-dimers and cryo-EM structures of complete virus capsids exist many open questions remain. For instance, the driving force for the "open"-"closed" transition has not been clearly identified yet. Based on solution NMR studies we hypothesize that the transition is linked to the stability of the dimeric form of P-domains in the viral capsid (Creutznacher et al. 2021a). A partial assignment of side chain ^13^C-methyl groups of MNV P-dimers has been instrumental for that study and will be reported in detail here. Initially, we had aimed at a backbone assignment as accomplished for a related human norovirus (HuNoV) P-dimer (Mallagaray et al. 2019) but it turned out that MNV P-dimers behave profoundly different. Consequently, we failed to identify an unfolding-refolding protocol with acceptable yields. Such a protocol would have been mandatory for the observation of slowly exchanging backbone NH protons and in turn for the successful application of 3D triple resonance NMR experiments for assignment. Side chain ^13^C methyl group labeling provides an alternative, also allowing to map ligand binding onto the protein surface and to study the exchange kinetics of ligand binding. Therefore, we prepared specifically ^13^C-methyl group labeled MNV P-dimer samples and employed a combination of 4D HMQC-NOESY-HMQC experiments, site-directed mutagenesis, and measurement of pseudocontact shifts for ^13^C-methyl group assignment. We succeeded in assigning ca. 70% of all Ala, Ile, Leu^ProS^, Met, and Val^ProS^ methyl groups.

## Methods and experiments

### Protein expression and purification

P-domain point mutants were created using site-directed mutagenesis with primers listed in Table S1 and confirmed by DNA sequencing.

[*U*-^2^H] ^13^C-methyl labeled MNV.CW1 P-domains (GenBank DQ285629, aa 228–530, *N*-terminal addition of GP peptide) as well as its point mutants were synthesized and purified as described elsewhere (Creutznacher et al. 2021a). In short, *E. coli* BL21 DE3 cells were transformed with a pMal-c2x expression vector encoding genes for ampicillin resistance and a fusion protein of maltose-binding-protein (MBP) and the P-domain, separated by two His_8_-tags and a HRV3C cleavage site. The proteins were expressed in D_2_O-based minimal medium using 3 g/L deuterated glucose (Deutero) as carbon source and 3 g/L ^14^NH_4_Cl (Deutero) as nitrogen source. Amounts of isotopically labelled precursors for [*U*-^2^H], ε-[^13^C,^1^H_3_]-Met (M), δ1-[^13^C,^1^H_3_]-Ile (I), γ2-[^13^C,^1^H_3_]-Val (V), δ2-[^13^C,^1^H_3_]-Leu (L), β-[^13^C,^1^H_3_]-Ala-labeling (A) of methyl groups are given in Table S2. For selective unlabeling of δ2-[^13^C,^1^H_3_]-Leu, 80 mg/L of L-leucine-d_10_ were added to the medium. One fifth of the final culture volume minimal medium was used to dissolve the respective precursors and the pH* (pH reading not corrected for ^2^H) was adjusted to 7.0. Cell growths was performed at 37 °C until an OD_600_ of 0.75 was reached. Afterwards, the solution containing the precursors was added and the culture was cooled down to 16 °C. Expression was induced by addition of 0.1 mM IPTG 1 h after adding the precursors. Growth was then continued at 16 °C until reaching the stationary phase. Cells were harvested by centrifugation and stored at − 80 °C.

For purification, cells were resuspended in a solution containing 20 mM sodium acetate and 100 mM NaCl (pH 5.3) and lysed using a high-pressure homogenizer (Thermo). The lysate was clarified by ultra-centrifugation. The fusion protein was purified performing Ni–NTA affinity chromatography and cleaved by addition of HRV3C protease. Subsequently, the protein was dialyzed against 20 mM sodium acetate buffer, 100 mM NaCl (pH 5.3). Ni–NTA affinity chromatography was repeated yielding the P-domain. Concentrated P-domain was subjected to gel filtration employing a preparative Superdex 16/600 200 pg size exclusion column (GE) using 20 mM sodium acetate, 100 mM NaCl (pH 5.3) as running buffer. Protein concentrations were determined using UV spectroscopy with a molar extinction coefficient of 46,870 M^−1^ cm^−1^. The proteins were concentrated using Amicon centrifugal filters (10 kDa, Merck) to their final concentrations. To achieve protein concentrations of 500 μM, the protein was concentrated in the presence of a tenfold molar excess of GCDCA.

### Sample preparation and NMR spectroscopy

All P-domain samples were prepared in D_2_O-based buffers, containing 20 mM sodium acetate-d_3_, 100 mM NaCl, 100 µM DSS-d_6_, (pH* 5.26), and saturating amounts of GCDCA to shift the monomer–dimer equilibrium exclusively towards dimers. Based on a dissociation constant of 6 μM (Nelson et al. 2018) a 1.4 molar excess as used in the 4D HMQC-NOESY-HMQC experiment (see below) leads to 97% saturation of GCDCA binding pockets. For the other samples a tenfold molar excess of GCDCA was used leading to almost 100% saturation. Samples were passed over Zebra™ Spin desalting columns (MWCO 7 kDa, Thermo Scientific) pre-equilibrated with the deuterated buffer and then transferred to 3 mm NMR tubes. EuCl_3_, SaCl_3_, CeCl_3_, LaCl_3_, and CaCl_2_ (Sigma-Aldrich) were titrated from highly concentrated stocks containing the respective salt, 20 mM sodium acetate d_3_, and 100 mM NaCl at a pH of 5.3.

Acquisition parameters for ^1^H,^13^C HMQC (methyl TROSY) spectra (Tugarinov et al. 2003; Ollerenshaw et al. 2003) are specified in corresponding figure legends. Additional experimental settings are compiled in Table S3. For acquisition of the 4D HMQC-NOESY-HMQC spectrum (Tugarinov et al. 2005), 30% non-uniform-sampling (NUS) was applied using a Poisson gap schedule (Hyberts et al. 2014, 2013) with 14087 NUS data points in a grid of 86 (^13^C) × 84 (^1^H) × 52 (^13^C) data points in the indirect dimensions. 512 data points were acquired in the direct dimension and a mixing time of 120 ms was used. The spectrum was reconstructed using the recursive MDD (multi-dimensional decomposition) algorithm as implemented with TopSpin 4.0.2 (Bruker).

All other NMR spectra were processed with TopSpin v3.6 (Bruker). For the analysis of ^1^H,^13^C HMQC spectra the CcpNmr Analysis v2.4.2 software suite (Vranken et al. 2005) was used.

## Extent of assignment and data deposition

### Amino acid type determination and assignments based on 4D HMQC-NOESY-HMQC

Attempts to obtain uniformly ^2^H,^15^N labeled samples of the MNV P-domain employing our published protocol for HuNoV GII.4 Saga P-dimers (Mallagaray et al. 2019) failed, yielding unstable protein samples as reflected by corresponding TROSY HSQC spectra (Creutznacher et al. 2021a). All attempts to obtain uniformly ^2^H,^13^C,^15^N backbone labeled MNV P-domain samples for performing a full backbone assignment have failed so far because we have not yet been able to identify an unfolding-refolding protocol that would allow exchange of slowly exchanging backbone N^2^H into NH at acceptable yields. At the size of the protein of ca. 70 kDa such a protocol is essential for successful backbone assignment (Mallagaray et al. 2019). Therefore, we proceeded with side chain ^13^C-methyl group labeling against a strongly deuterated background. This approach has been shown to be of advantage especially for studies of large proteins (Ruschak and Kay 2010; Velyvis et al. 2009a; Velyvis et al. 2009b; Sprangers and Kay 2007; Tugarinov et al. 2006; Tugarinov et al. 2005; Schutz and Sprangers 2020; Cvetkovic and Sprangers 2018; Wiesner and Sprangers 2015), and we recently applied it to HuNoV GII.4 Saga P-dimers where a full assignment of methyl groups of Ala, Ile, Leu^proS^, Met, and Val^proS^ (MIL^proS^V^proS^A, in the following this will be abbreviated "MILVA") residues was possible based on methyl-methyl NOEs and point mutants (Müller-Hermes et al. 2020). For the MNV P-domain the choice of an acidic pH was critical for obtaining stable samples. Furthermore, MNV P-domains in solution are an equilibrium of monomers and dimers, with significant amounts of monomers present at room temperature (Creutznacher et al. 2021a). In contrast, HuNoV P-domains form very stable dimers. In that study (Creutznacher et al. 2021a) we also show that GCDCA significantly stabilizes the dimeric state of MNV P-domain, and at the same time induces conformational changes in loop regions. The monomer–dimer equilibrium leads to spectral crowding and impedes structure-based assignment of methyl-methyl NOEs. Therefore, 4D HMQC-NOESY-HMQC experiments were performed in the presence of saturating amounts of GCDCA, shifting the monomer–dimer equilibrium almost exclusively towards dimers. Likewise, all other NMR experiments aiming at assignment of ^13^C-methyl group resonance signals have been performed with MNV P-dimers stabilized by GCDCA.

**Fig. 1:**
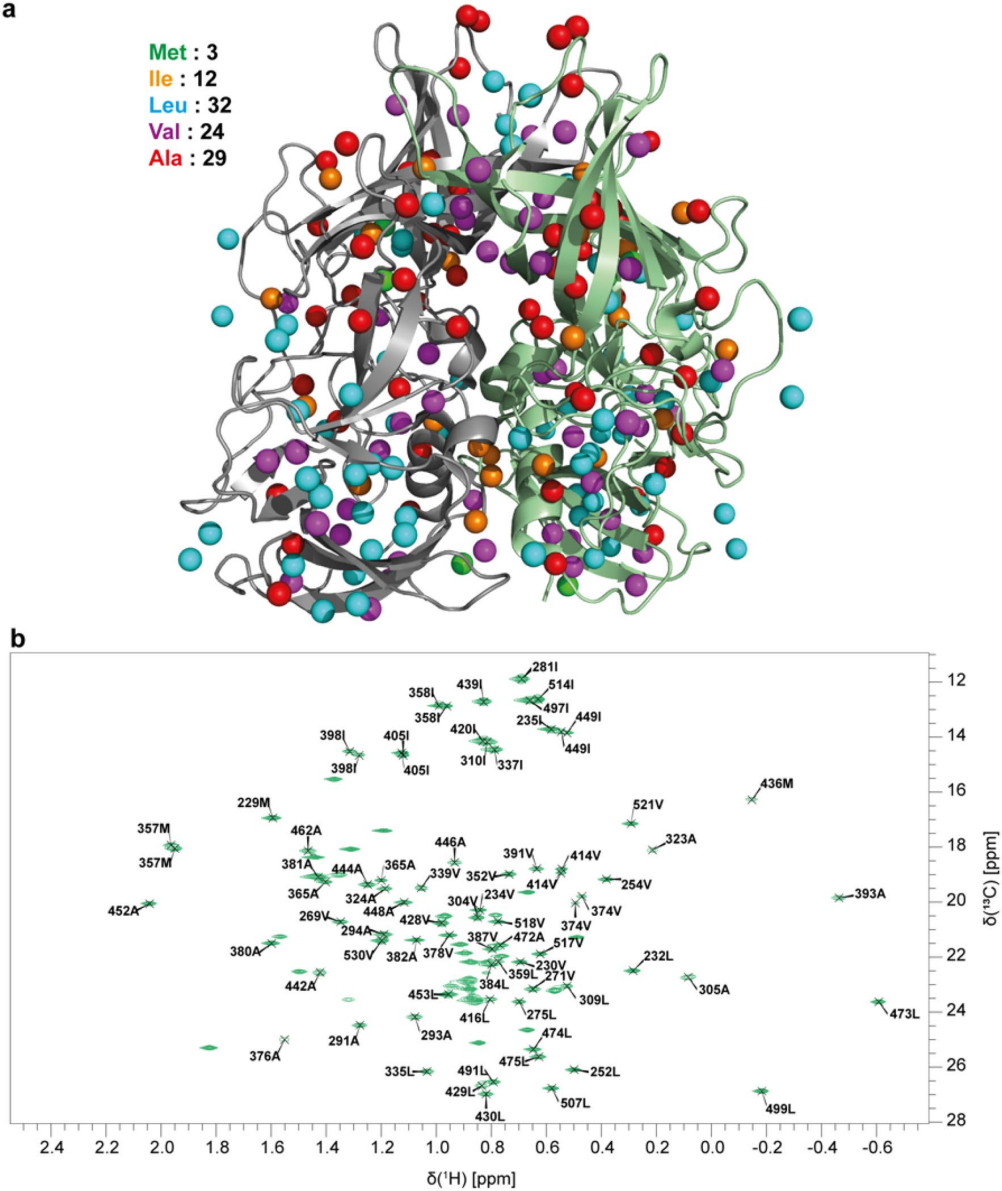
Assignment of methyl TROSY cross peaks of MILVA ^13^C-methyl labeled MNV P-dimers. **a** Structural model (pdb 6e47) of the MNV P-dimer with MILVA methyl groups color coded. **b** Partial assignment of MILVA methyl groups of MNV P-dimer. The sample contained 500 µM MILVA P-domain and saturating amounts of GCDCA. The spectrum was recorded on a Bruker 600 MHz Avance III HD spectrometer equipped with a TCI cryogenic probe at 298 K

As will become clear in the following, full assignment of MILVA methyl groups of MNV P-dimers solely based on methyl-methyl NOEs is impossible. Point mutations and measurement of pseudo contact shifts (PCS) have been instrumental for an assignment of better than 55%. Assignments based on methyl-methyl NOEs or on PCSs require the availability of an appropriate structural model (Proudfoot et al. 2016; Flügge and Peters 2018; Xiao et al. 2015; Venditti et al. 2011; Pritišanac et al. 2020). To this end, we have used coordinates from a high-resolution crystal structure of MNV P-dimers complexed with GCDCA (Nelson et al. 2018). It should be noted that the CW3 strain used in that study differs in one amino acid from the CW1 strain used here, i.e., K296 in CW3 vs. E296 in CW1. For structure-based assignments this difference can be ignored. The homodimer has 200 MILVA methyl groups, theoretically resulting in 100 cross peaks in a methyl TROSY spectrum of the P-dimer, given the monomers are symmetrically arranged. In fact, superposition of the two monomers forming the P-dimer in the crystal structure (pdb code: 6e47, cf. Fig. 1a) leads to a RMSD value of 1.52 Å for the Cα positions. Surprisingly, 103 instead of 100 methyl group resonances are observed in the corresponding methyl TROSY spectrum (Fig. 1b).

Methyl TROSY spectra of selectively A, LV, V, ILV and MI ^13^C methyl group labeled MNV P-domain samples were compared to the corresponding methyl TROSY spectra of the MILVA labeled sample to identify amino acid types. We found 29 of 29 expected alanine resonances, 28 of 32 expected leucine resonances, 16 of 12 expected isoleucine resonances, 4 of 3 expected methionine resonances, and 26 of 24 expected valine resonances (Fig. S1 a–c). Whereas missing methyl resonances can be explained by factors increasing transverse relaxation such as, e.g., incomplete background deuteration (Proudfoot et al. 2016) the occurrence of additional signals may indicate an erroneous coding gene sequence. This possibility has been excluded by sequencing of the gene construct used and suggests that different forms of the protein exist in solution.

Structure-based analysis of a 4D HMQC-NOESY-HMQC spectrum (Tugarinov et al. 2005) of a MILVA labeled MNV P-domain sample using the methyl-walk strategy allowed unambiguous assignment of 55 methyl groups. Briefly, cross peaks in F1(^1^H)/F2(^13^C) planes (Fig. S2a) with the F3(^13^C)/F4(^1^H) frequencies set at the ^13^C and ^1^H chemical shifts of a specific methyl group resonance (Fig. S2 c) are either auto peaks appearing at F1 = F4 and F2 = F3, e.g., the peak labelled Ala382 in the first panel of Fig. S2 a), or they are cross peaks reporting NOEs between methyl groups. Using the information about amino acid types and structural restraints from the crystal structure (Fig. S2b) it is possible to assign the NOEs to distances between specific methyl groups. Repeatedly moving on to the next F1/F2 planes (red dashed lines in Fig. S2a) and assignment of the corresponding NOEs to neighboring side chain methyl groups theoretically leads to a complete assignment. In practice, gaps occur that require application of complementary assignment techniques.

### Point mutations and titration with metal ions enable assignment of methyl groups in loop regions and expose two configurations of the P-domain

**Fig. 2:**
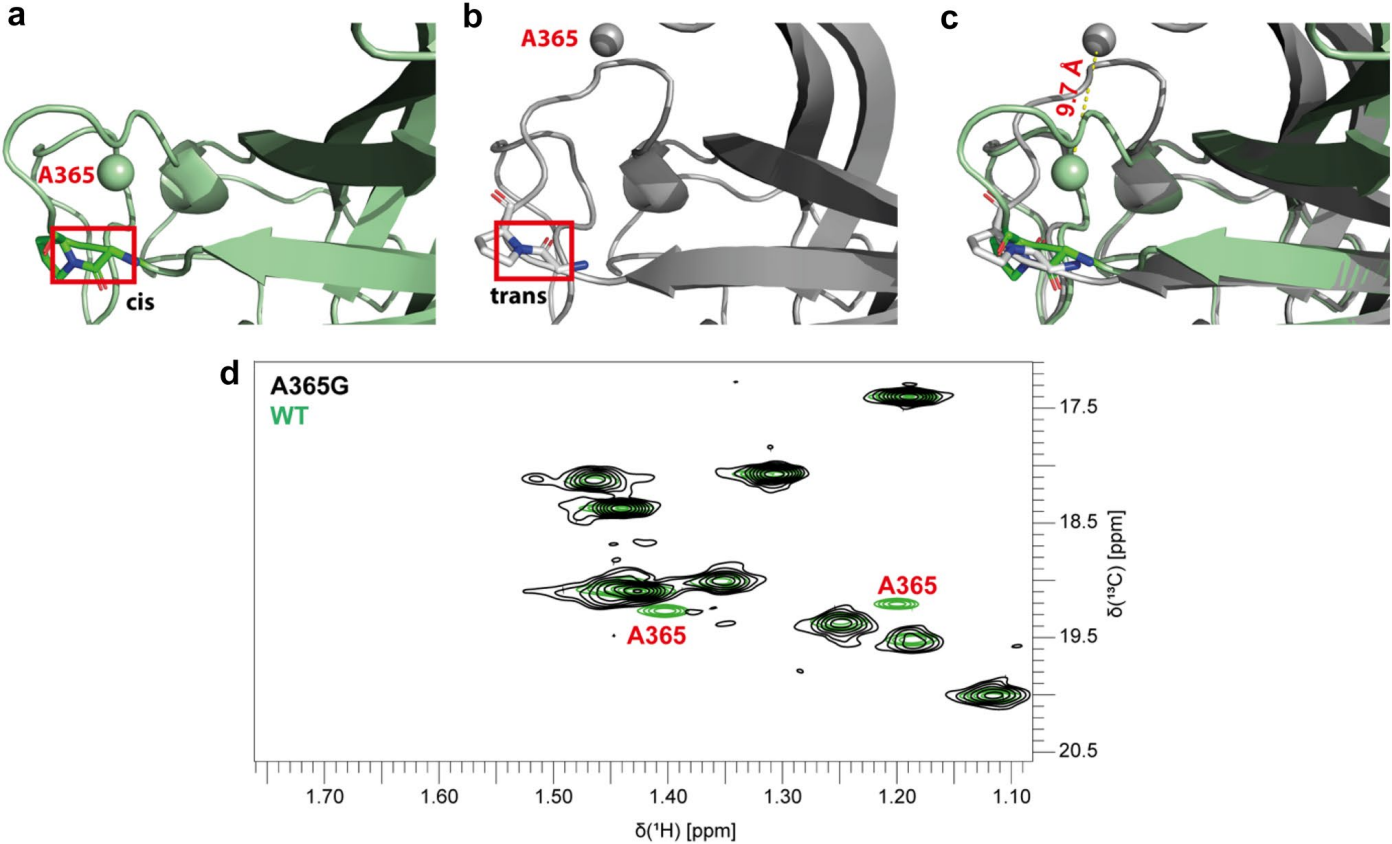
MNV P-dimers are a mixture of trans-P361 and cis-P361 isomers. **a**, **b** A and B chain of the P-domain of MNV as found in a crystal structure model (Koromyslova et al. 2020) (pdb 6xw5). The red box indicates the peptide bond between G360 and P361. It is obvious that the neighboring loop containing A365 has different conformations, depending on the configuration of the G360-P361 peptide bond. In **c** an overlay of the cis and the trans loop conformations is shown. The A365 methyl group positions differ by ca. 10 Å. **d** The superposition of ^1^H,^13^C HMQC spectra of wild-type (green) and A365G mutant (black) P-dimers of MNV shows two peaks disappearing upon mutation, allocating both peaks to A365. The splitting into two peaks of about equal intensities reflects the presence of a mixture of trans-P361 and cis-P361 isomers. The spectrum of A365G was acquired with 45 µM A-labelled sample of A365G P-dimers. For details of the spectrum of wild-type P-dimers see Fig. S2. Both spectra were acquired at 298 K on a Bruker Avance III HD 600 MHz spectrometer equipped with a cryogenic probe

The methyl-methyl NOE based assignment leaves a substantial number of methyl groups unassigned (Fig. S2 d). One approach to fill these gaps was to study selected point mutants. Eleven single point mutations were generated (Table S1) and methyl TROSY spectra were compared to the corresponding wild-type spectra. To save on measurement time we combined A, I, and LV labeled protein in single NMR samples. Combination of mutants were chosen such that no signal overlap was possible (Table S3). Five mutants, V304L, A442G, V387L, V378L and A444G allowed unambiguous assignments based on the absence of single resonances in comparison to wild-type P-domain (Fig. S3 a–f). Assignment of A381 via the A381G point mutant matched the assignment obtained from the methyl walk. To our surprise, for five mutants, V352L, I358L, A365G, I405L, and A446G two signals disappeared in comparison to the wild-type methyl TROSY spectra (Fig. S3 h–k and Fig. 2d). In the case of V352L and A446G we had independent unambiguous assignments for one of the two signals disappearing. For V352L the second peak has been identified as V391 cross peak based on methyl-methyl NOEs (Fig. S4). For A446G the second peak belongs to A448G as derived from PCS (see below). Therefore, in these two cases altered transverse relaxation properties because of incomplete deuteration or changed protein dynamics (Proudfoot et al. 2016) likely account for the V391 signal disappearing. It is also possible that the corresponding peaks shift to a position that cannot be identified because of signal overlap. At this point, it should be highlighted that single point mutations affect the chemical shifts of remote amino acids to a varying degree, likely containing information on long-range interactions (Aoto et al. 2016). For point mutants I358L and A365G the additional peaks disappearing had been unassigned. For I405 an assignment was available from NOEs, and the point mutation served as a control. We inferred that these peaks represent a second form of the protein. To test this hypothesis, we performed control experiments, e.g., titrations with metal ions as described in the following.

For the MNV P-dimers, two binding sites for bivalent metal ions per monomer exist (Nelson et al. 2018) (Fig. S5 a). Following ^13^C-methyl group chemical shift perturbations (CSPs) through a CaCl_2_ titration revealed two sets of signals, a first set including Ala442 and Ala444 with CSPs reaching saturation at ca. 550 μM CaCl_2_, and a second set containing Ala365 and Ile358, approaching saturation at ca. 20 mM CaCl_2_ (Figs. S5 b,c and d,e). The two sets of resonance signals match well with the methyl groups in the two metal ion binding loops as found in the crystal structure (examples shown in Figs. S5 a–e, for assignment of the methyl groups see below). It turns out the peak pairs in question behave alike in these titration experiments, supporting the hypothesis that they belong to two different forms of the protein.

We then tested binding of lanthanide ions. Titration with LaCl_3_ yielded similar titration curves as with CaCl_2_ discriminating the two metal ion binding sites. However, the pairs of cross peaks of the residues belonging to the low-affinity site, i.e., Ala365, Ile358, and Ile405 showed distinct behavior. Only one peak of each pair shifted continuously with increasing LaCl_3_ concentrations as this would be expected for fast chemical exchange usually associated with low-affinity binding. The other cross peak disappeared at saturating La^3+^ concentrations in the low mM range, indicating a slow exchange process affecting this signal and highlighting that the two postulated forms of the protein respond differently to La^3+^ binding (Fig. S6c). Inspection of methyl TROSY spectra of MNV P-dimers at saturating La^3+^ concentrations revealed five other amino acids, Met357, V374, I398, V414, and I449 that are associated with two methyl cross peaks each (Fig. S5b). These methyl groups had been unambiguously assigned based on methyl-methyl NOEs. Close inspection of the corresponding cross peaks in methyl TROSY spectra shows that each peak consists of two closely overlapping peaks. This becomes very clear when comparing to methyl TROSY spectra at saturating La^3+^ concentrations where only one of the peaks is left because of severe line broadening of the other peak, essentially displaying the same behavior as A365, I358 and I405 for which point mutations had shown that the double peaks belong to the same amino acid. Therefore, we classified these eight amino acids as representing two distinct forms of the MNV P-dimers, present at approximately equal amounts.

To identify the cause for the occurrence of double peaks we tested different hypotheses. Exchange between two conformations was excluded as the relative amount of each form was insensitive to temperatures ranging from 298 to 323 K. The possibility of a spontaneous post-translational modification appeared as another potential explanation. However, such modifications, e.g., spontaneous asparagine deamidation (Mallagaray et al. 2019) proceed towards the final product and would not explain the occurrence of two forms at about equal concentrations independent of the age of the sample. Therefore, if a post-translational modification is causing the occurrence of two species at a set ratio the underlying reaction must come to a stop before the sample is subjected to, e.g., NMR experiments. A modification that may fulfill this requirement is cysteine side chain oxidation and subsequent rearrangement reactions. An unpaired Cys residue may be exposed, e.g., in a monomeric form of the P-domain during protein synthesis, and only after workup and purification that residue would be buried in the dimer. Crystal structure models suggest that Cys288 may be exposed in a monomeric form of the protein. Therefore, we subjected P-dimers to mass spectrometry-based peptide mapping employing different sets of proteases (for details see supporting information). No clear indication of any post translational backbone modification was found. Only for peptides containing Cys288 ~ 20% showed a mass difference of − 2 u, compatible with, e.g., formation of a sulfenylamide (Fig. S7). However, this does not explain the approximately equal distribution of the two forms observed in NMR spectra. Dilution of NMR samples to low μM concentrations to increase the monomeric form of the P-dimers and treatment with reducing as well as oxidizing agents did not alter the appearance of methyl TROSY spectra recorded after reconcentrating the samples and adding GCDCA. Therefore, we abandoned the possibility of post translational backbone modifications as a cause for the two sets of signals.

Inspection of crystal structure models of MNV P-domains shows that Pro361 in some structures is found in the preferred *trans* configuration, e.g., in (Taube et al. 2010) whereas in other structures, e.g., in (Nelson et al. 2018) P361 adopts the *cis* configuration. In one structure model both configurations are present in the same P-dimer (Koromyslova et al. 2020) (Fig. 2a–c). This shows that protein synthesis of MNV P-domains yields a mixture of species, one with Pro361 in the *cis* and one in the *trans* configuration. In other cases, the protein crystallizes preferentially in one of the two forms yielding crystals with either *trans* or *cis* configured species. In solution both configurations are present and give rise to distinct NMR spectra. The switch of Pro361 from *trans* to *cis* is associated with a substantial reorientation of the subsequent surface loop containing Ala365, where the chemical shift difference between the two cross peaks in methyl TROSY spectra is most pronounced (Fig. 2d). All eight amino acids (Fig. S6a) for which double peaks have been identified are located within ca. 15 Å of Pro361. The effects of changing the configuration of proline residues in proteins on NMR spectra has been observed and discussed before but reports of proteins that come in both "flavors" at the same time are rare (Kawagoe et al. 2018; Alderson et al. 2017; Roderer et al. 2015; Chazin et al. 1989).

### Assignments using pseudo contact shifts

Alignment tensor parameters and the location of the paramagnetic center were fitted assuming a single paramagnetic center as described in detail below. The fitting was performed with *Paramagpy* (Orton et al. 2020) in two steps. In a first step, ΔΧ_ax_ and ΔΧ_rh_, α, β and γ, and the coordinates x, y, and z of the paramagnetic center were fitted. In a second step, x, y, and z coordinates were kept constant, and ΔΧ_ax_ and ΔΧ_rh_, α, β and γ were fitted. Error analysis employed *Paramagpy*’s built-in bootstrap algorithm using 90% of the data (default setting) and 1000 iterations.

To extend the assignment and to validate assignments from methyl-methyl NOEs and single point mutations we measured pseudo contact shifts (PCSs). Assignments were based on a high-resolution crystal structure (pdb 6e47) of MNV P-dimers (Nelson et al. 2018) following established protocols for the back calculation of PCSs from structural data (Orton et al. 2020; Otting 2010). There are two binding sites for bivalent metal ions per monomeric unit, to which also lanthanide ions can bind. In a previous study we have determined the dissociation constant for Ca^2+^ as 138 μM based on CSPs measured for unassigned cross peaks in ^1^H,^15^ N HSQC TROSY spectra (Creutznacher et al. 2021b). Here, we repeated a titration with Ca^2+^ (CaCl_2_) following CSPs in methyl TROSY spectra (Fig. S5). One set of peaks yields binding isotherms going into saturation at Ca^2+^ concentrations around 0.55 mM (Fig. S5b), confirming the order of magnitude of the previously measured dissociation constant. However, a second set of cross peaks (Fig. S5d) leads to binding isotherms approaching saturation at much higher concentrations of Ca^2+^ around 20 mM, indicating a dissociation constant in the mM range. We conclude that these two types of binding isotherms reflect the binding sites known from crystallography. For PCS-based assignments we assume that the dissociation constants for lanthanide ions are comparable. Therefore, using concentrations of lanthanide ions around 400 μM will saturate the higher-affinity metal ion binding pocket but not the low affinity one.

Experimental ^1^H PCSs induced by Ce^3+^, Eu^3+^, and Sm^3+^ were the basis for the structure-based assignment. We acquired methyl TROSY spectra of MILVA-labeled P-domain in the presence of CeCl_3_, EuCl_3_ and SmCl_3_. LaCl_3_ was used as diamagnetic reference (Fig. S8 b). The metal ion binding sites of the P-dimers are symmetrically arranged. Therefore, determination of the alignment tensor and locating the paramagnetic centers requires editing of the crystal structure model. To fit tensors using a single paramagnetic center located in one monomeric unit, we first compared distances of each atom in one monomer, called the B chain, to the metal ion of the same monomer (distance d_1_ and metal ion B in Fig. S8 a) to the respective distances of atoms in the A-chain to the metal ion B (distances d_2_ in Fig. S8 a). For further calculations, we only kept those atoms of either A or B chain having the smaller distance d_1_ or d_2_. This leads to an edited structure model as shown in the lower panel of Fig. S8 a.

**Fig. 3:**
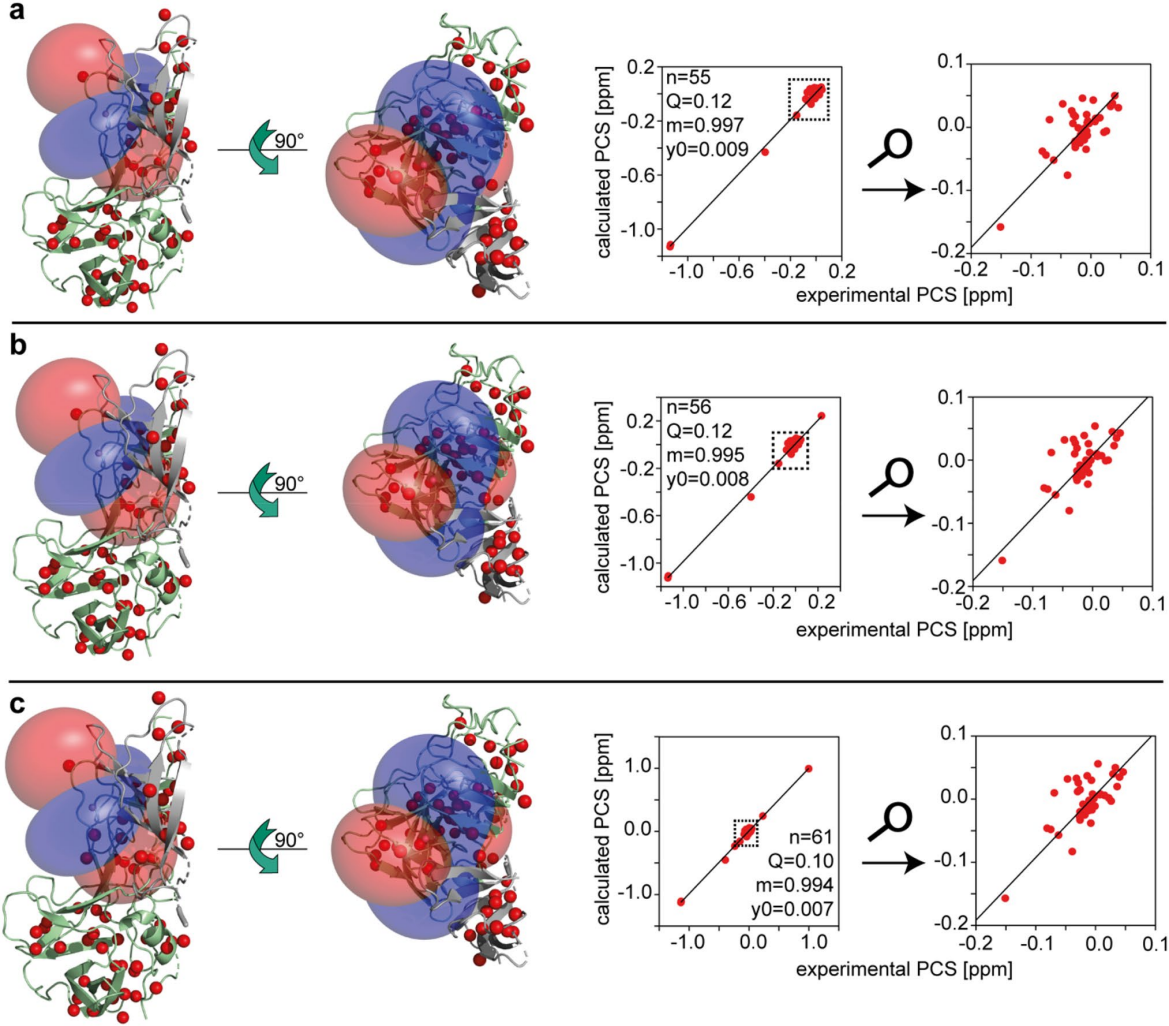
Assignment of ^13^C methyl group resonances of MNV P-dimers using PCSs. **a** Using experimental PCSs of 55 methyl groups assigned via methyl-methyl NOEs (Fig. S2) and point mutations (Fig. S3) the alignment tensor for Ce^3+^ was determined (Table 1, "first round"). **b** In a second round of fitting Ile439 was included (Table 1, "second round") leading to a better-defined γ-angle. (c) A third round of fitting included the assignments of the methyl groups of Ala446, Ala448, Ile514, Ile310, and Val234 yielding a well-defined γ-angle and an improved Q-factor of 0.10 (Table 1). For details of the assignment process see the main text

Fitting the position of the paramagnetic center and parameters of the alignment tensor of Ce^3+^ using PCSs of 55 of the 63 assigned methyl groups resulted in a reasonable Q-factor of 0.12 with the paramagnetic center located close to the position of metal ion B in the structure model (Table 1 and Fig. 3a). The γ angle of is not well defined yet (Table 1). Nevertheless, this alignment tensor allows unambiguous assignment of one of the unassigned isoleucine methyl group resonances as Ile 439 (Fig. S9). Ile 439 is the only isoleucine residue that is inside a sphere of 20 Å around the higher-affinity metal ion binding site. This assignment is supported by the observation of small CSPs upon addition of Ca^2+^ ions (Fig. S9b). Including Ile 439 in a second round of fitting, similar alignment tensor parameters are obtained for Ce^3+^ (Table 1 and Fig. 3b). However, the γ angle is much better defined now (Table 1). Therefore, we used theoretical PCSs based on this tensor combined with additional information to obtain further assignments.

**Table 1:** Parameters from alignment tensor fitting for Ce^3+^

	First round	Second round	Third round
ΔΧ_ax_ in			
10^–32^ m^3^	− 1.60 ± 0.05	− 1.60 ± 0.05	− 1.60 ± 0.05
ΔΧ_rh_ in			
10^–32^ m^3^	− 0.66 ± 0.07	− 0.69 ± 0.07	− 0.74 ± 0.04
Coordinates of origin in Å			
X	− 16.4 ± 0.9	− 16.6 ± 0.8	− 16.3 ± 0.5
Y	2.8 ± 1.4	2.9 ± 0.7	2.9 ± 0.4
z	− 44.9 ± 1.4	− 45.4 ± 1.1	− 45.3 ± 0.7
Orientation of principal axis of tensor in °			
α	109.8 ± 0.7	107.5 ± 1.0	109.6 ± 0.3
β	66.0 ± 3.2	64.9 ± 3.1	66.8 ± 2.4
γ	168.6 ± 18.8	155.6 ± 4.1	155.9 ± 2.0
Amount of methyl groups included	55	56	61
Q-factor	0.12	0.12	0.10
Distance to metal ion position in the crystal structure in Å^a^	2.2	2.1	1.9

^a^For errors of the position of the paramagnetic center, see row for coordinates of origin. The resolution of the crystal structure is 2 Å

Assignment of A446 by comparing spectra of A-labelled A446G and wild-type P-domain failed since two resonance signals disappeared due to the point mutation (Fig. S10 a). Both resonances must belong to the loop forming the higher affinity metal binding site (Fig. S10 c). In this loop, three unassigned alanine methyl groups are left (Fig S10 b). Theoretical Ce^3+^-induced PCSs are as follows: 0.776 ppm for Ala446, 1.651 ppm for Ala 445, and − 0.237 ppm for Ala448. The PCS values measured for the two resonances that disappear upon mutation are 0.997 ppm and − 0.237 ppm (Fig. S10 d). Therefore, the resonances at 1.12 ppm (^1^H) / 20.1 ppm (^13^C) and at 0.93 ppm (^1^H)/18.6 ppm (^13^C) were assigned to Ala448 and to Ala446, respectively.

For the isoleucine methyl groups I310 and I514 PCSs of 0.034 ppm and of − 0.012 ppm were calculated (Fig. S11 a). These values match well with the experimental PCSs of − 0.009 ppm and of 0.029 ppm for the two remaining unassigned Ile cross peaks (Fig. S11 b). Assignment of Ile514 closes a gap in the assignment of Val methyl group cross peaks: a NOE cross peak between the methyl group of Ile514 and a so far unassigned Val methyl group identifies this methyl resonance as Val234 (Fig. S11 c,d). Including the additional assignments in a third round of tensor fitting yielded an improved Q-factor and well-defined tensor parameters (Table 1 and Fig. 3c). The position of the paramagnetic center (Ce^3+^) was very close to the metal ion (Mg^2+^) site as found in the crystal structure (Table 1).

To validate the assignments, we compared theoretical and experimental PCSs caused by Sm^3+^ and Eu^3+^. We used the edited structural model as described above to calculate tensor parameters and the position of the paramagnetic center. To analyze the influence of the size of the two distances d_1_ and d_2_ of a given methyl group to the two symmetry-related metal binding sites on the fitting process we gradually excluded PCSs of methyl groups that displayed similar distances d_1_ and d_2_. We used the difference between the two distances |d_1_–d_2_| as an exclusion criterion (Fig. S12). One round of fitting was done using no restrictions, i.e., |d_1_–d_2_|> 0 Å (Fig. S12 b.i). A second round of fitting applied |d_1_–d_2_|> 2.5 Å (Fig. S12 ci) as a filter, and a third run employed |d_1_–d_2_|> 5 Å (Fig. S12 di). It is obvious and maybe not surprising that progressively excluding PCSs associated with similar distances to the two symmetry-related metal ion binding sites improves Q-factors (Fig. S12 b–d, ii–iv).

Finally, 77 of 103 resonances were assigned to 69 of 100 methyl groups (Fig. 1b). This assignment is a basis for studies into the details of the dimerization process of MNV P-domains, and may help to understand how this dimerization is modulated by external factors such as bile acids, metal ions, pH and interactions with host proteins.

## Supplementary Information

Below is the link to the electronic supplementary material.

Supplementary file1 (DOCX 5079 KB)

## Data Availability

The datasets generated during and/or analyzed during the current study are available from the corresponding author on reasonable request. The resonance assignments were deposited with the BioMagResBank (https://www.bmrb.wisc.edu) under accession number 50919.
