# Supplementary material for: Assignment of Ala, Ile, LeuproS, Met, and ValproS methyl groups of the protruding domain of murine norovirus capsid protein VP1 using methyl–methyl NOEs, site directed mutagenesis, and pseudocontact shifts
Source: Biomol NMR Assign. 2022 Jan 20;16(1):97–107. doi: 10.1007/s12104-022-10066-7 (PMC9068638; doi:10.1007/s12104-022-10066-7)
Supplement: Supplementary file 1 — Supplementary file1 (DOCX 5079 KB) [file 12104_2022_10066_MOESM1_ESM.docx]

**Supplementary Information**

**Assignment of Ala, Ile, Leu^proS^, Met, and Val^proS^ methyl groups of the protruding domain of murine norovirus capsid protein VP1 using methyl-methyl NOEs, site directed mutagenesis, and pseudocontact shifts**

Thorben Maass^1^, Leon Torben Westermann^1^, Robert Creutznacher^1^, Alvaro Mallagaray^1^, Jasmin Dülfer^2^, Charlotte Uetrecht^2,3^, and Thomas Peters^1^*

^1^ University of Luebeck, Center of Structural and Cell Biology in Medicine (CSCM), Institute of Chemistry and Metabolomics, Ratzeburger Allee 160, 23562 Luebeck, Germany

^2^ Leibniz Institute for Experimental Virology (HPI), 20251 Hamburg, Germany

^3^ School of Life Sciences, University of Siegen, 57076 Siegen & Centre for Structural Systems Biology (CSSB), & Deutsches Elektronensynchrotron (DESY), 22607 Hamburg & European XFEL GmbH, 22869 Schenefeld, Germany

Email corresponding author: [thomas.peters@uni-luebeck.de](mailto:mallagaray@chemie.uni-luebeck.de)

**Table of Contents**

[Fig. S1: Superposition of methyl TROSY spectra of a specifically ^13^C-methyl labeled samples of MNV P-dimers. 3](#_Toc87767776)

[Fig. S2: Structure based assignment of MILVA methyl group resonances of MNV P-dimers using a 4D HMQC-NOESY-HMQC spectrum and the methyl walk procedure. 4](#_Toc87767777)

[Fig. S3: Methyl TROSY spectra of MNV P-dimers and selected point mutants. 6](#_Toc87767778)

[Fig. S4: Assignment of Val391 using methyl-methyl NOEs. 7](#_Toc87767779)

[Fig. S5: CaCl_2_ titration attests two Ca^2+^ binding sites per monomer. 8](#_Toc87767780)

[Fig. S6: Titration with La^3+^ discloses two distinct forms of the MNV P-domain. 9](#_Toc87767781)

[Fig. S7: MS-based peptide mapping of 285-VSCFAAEAAY-296 of CW1 P Dimer 10](#_Toc87767782)

[Fig. S8: Preparing a crystal structure model for tensor fitting with Paramagpy. 11](#_Toc87767783)

[S9: Assignment of Ile439. 12](#_Toc87767784)

[S10: Assignment of Ala446 and Ala448. 13](#_Toc87767785)

[S11: Assignment of Ile514, Ile310 and Val234. 14](#_Toc87767786)

[S12: Validation of assignment using PCSs of Ce^3+^, Eu^3+^ and Sa^3+^. 15](#_Toc87767787)

[Tab. S1: Primers used for site-directed mutagenis. 16](#_Toc87767788)

[Tab. S2: Final concentrations of precursors for MILVA-labeling of MNV-P-domains. 17](#_Toc87767789)

[Tab. S3: ^1^H, ^13^C HMQC acquisition parameters. 18](#_Toc87767790)

[Tab. S4: Parameters of alignment tensors of Ce^3+^, Eu^3+^ and Sa^3+^ using different structural processing approaches (see Fig. S10). 19](#_Toc87767791)

[Supplemental information for mass spectrometry-based peptide mapping of the MNV P‑domain 20](#_Toc87767792)

[References 21](#_Toc87767793)

**
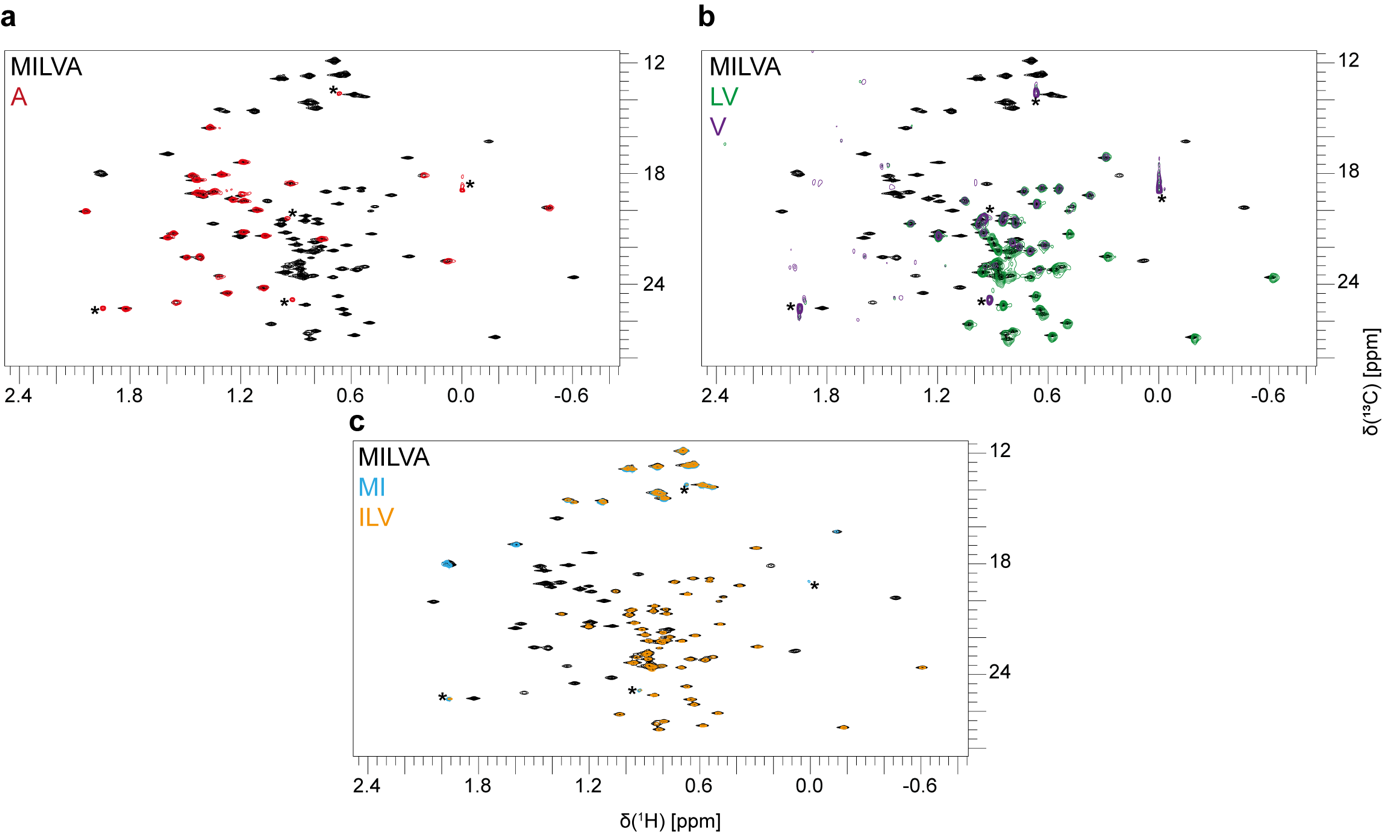
**

### Fig. S1: Superposition of methyl TROSY spectra of a specifically ^13^C-methyl labeled samples of MNV P-dimers.

Superposition of methyl TROSY spectra of a MILVA (500 µM) labeled sample of MNV P-dimers **(a-c)** with an ILV-labeled (50 µM) and a MI-labeled (40 µM) sample **(c)**, with a LV-labeled (4 µM) and a V‑labeled (2 µM) sample **(b)**, and with an A-labeled sample (7 µM) **(a).** The overlay allows counting the number of methyl group resonances for each amino acid type: 4 Met, 16 Ile, 28 Leu, 26 Val, and 29 Ala. All spectra except the one for the MI-labeled sample were recorded on a Bruker 600 MHz Avance III HD spectrometer equipped with a TCI cryogenic probe at 298 K. The MI-labeled sample was measured on a Bruker 500 MHz Avance III spectrometer equipped with a TCI cryogenic probe. Asterisks mark signals from GCDCA and acetate.


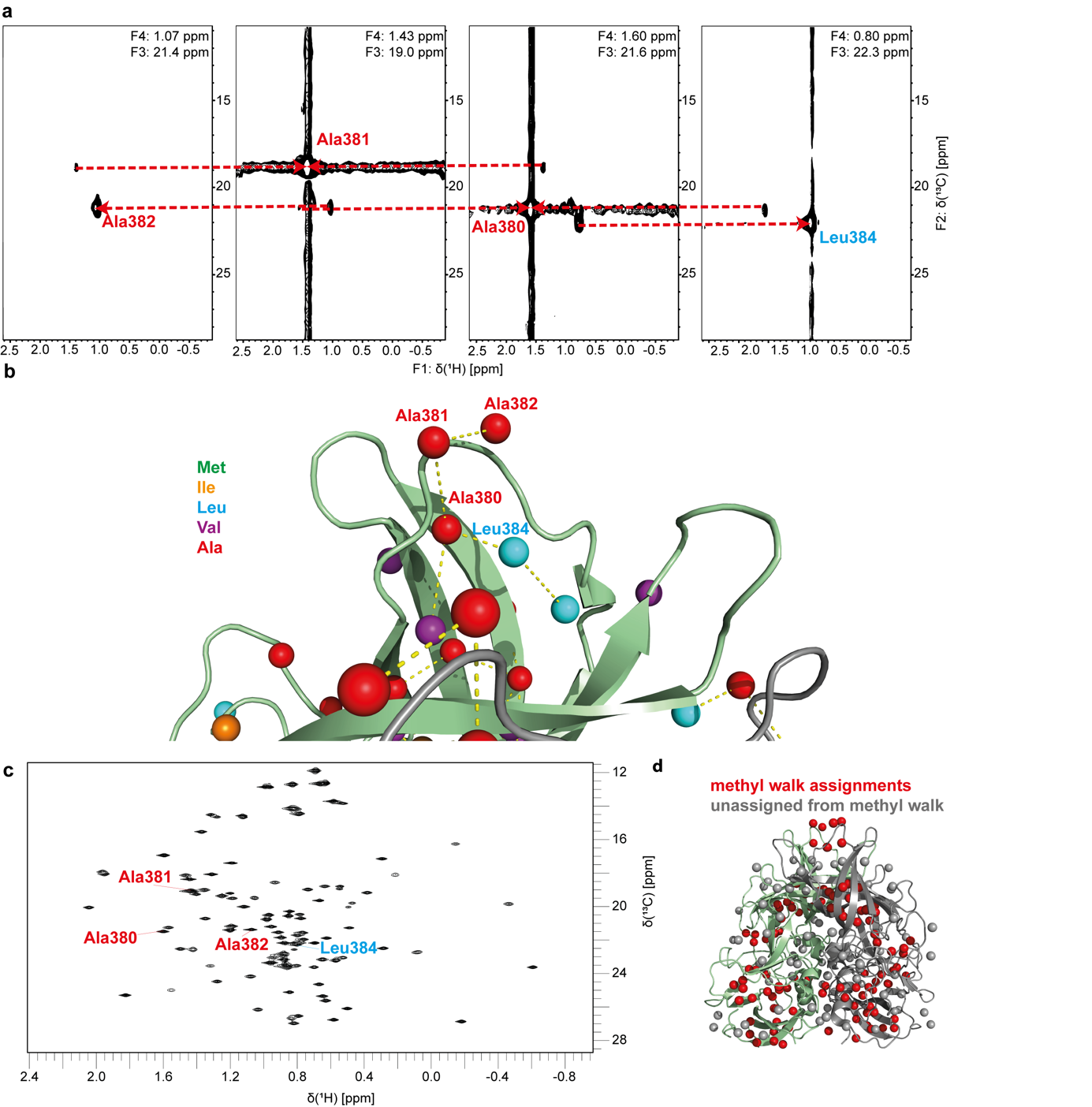


### Fig. S2: Structure based assignment of MILVA methyl group resonances of MNV P-dimers using a 4D HMQC-NOESY-HMQC spectrum and the methyl walk procedure.

**(a)** Structural restraints from methyl-methyl NOEs observed in F1-F2 planes of a 4D HMQC-NOESY-HMQC spectrum are systematically compared to methyl-methyl distances obtained from a high-resolution crystal structure **(b)** (pdb 6e47) until distance restraints from NOEs match distances found in the crystal structure. This process is called methyl walk, leading to unambiguous assignment of 55 methyl groups. Section **(a)** shows the methyl walk from A382 to L384 as an example. **(c)** Methyl TROSY spectrum of MNV P-dimers with the methyl group resonances of the amino acids in the methyl walk example highlighted. **(d)** methyl groups assigned via the methyl walk are red and those left unassigned by this procedure are grey. Spectra were acquired at 298 K on a Bruker Avance III HD 600 MHz spectrometer equipped with a cryogenic probe. The sample contained 500 µM MILVA-labeled MNV P‑domain at saturating concentrations of GCDCA.


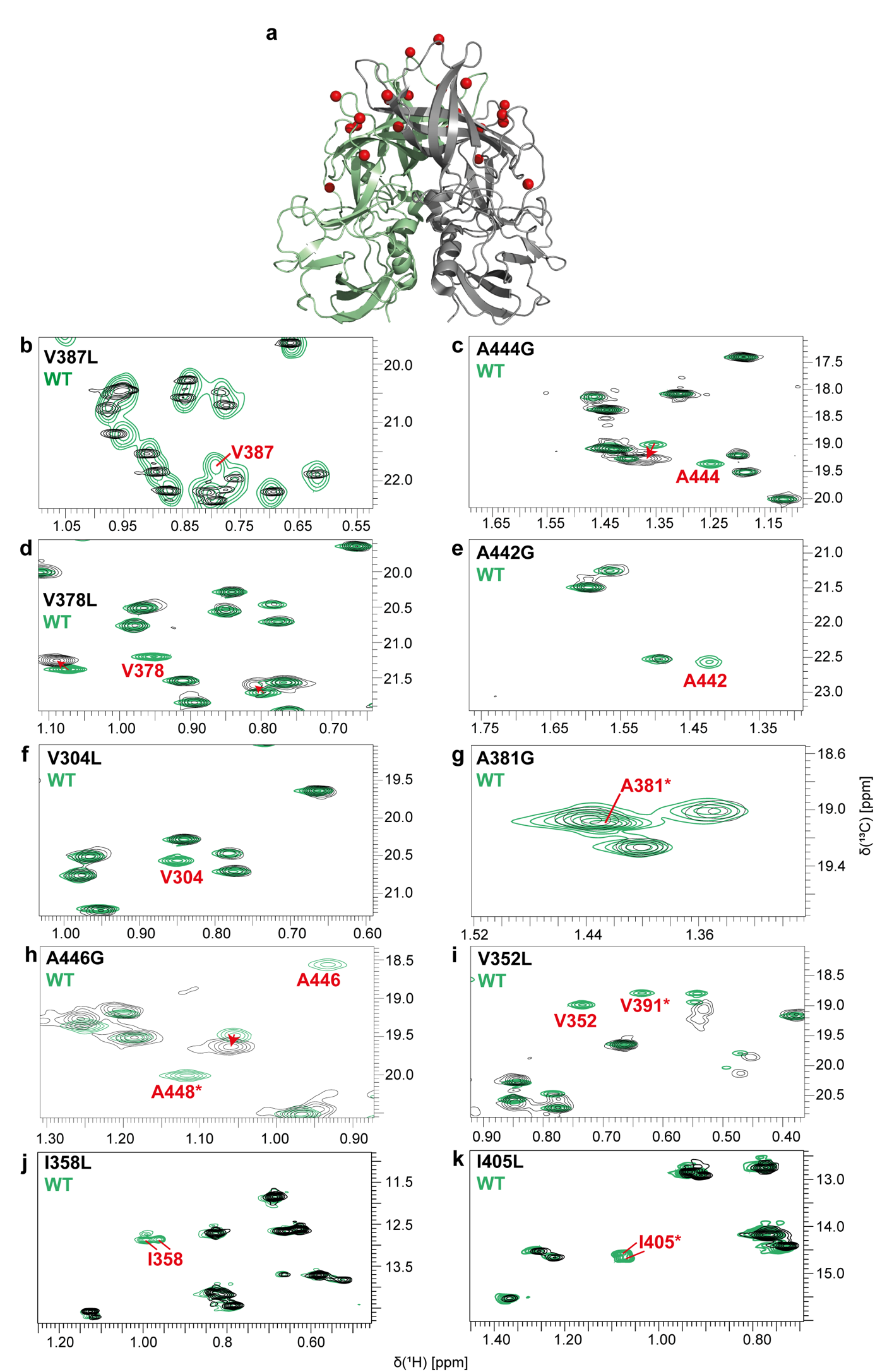


### Fig. S3: Methyl TROSY spectra of MNV P-dimers and selected point mutants.

Overlay of methyl TROSY spectra of wild type MNV P-dimers (green) with spectra of point mutants (black) yields an assignment of the mutated positions. All spectra were obtained in the presence of saturating amounts of GCDCA to shift the monomer-dimer equilibrium exclusively towards the dimers. **(a)** Single point mutations mapped onto the crystal structural model of MNV P-dimers (pdb 6e47). **(b‑f)** Comparison of spectra yielded the unambiguous assignment of methyl group resonances of V387, A444, V378, A442, and V304. **(g)** The methyl group of A381 had already been assigned via the methyl walk (see Fig. S2). Therefore, the rather similar cross peak patterns of wild type P‑dimer and of its A381G mutant imply overlap with another resonance. **(h, i)** For A446G and V352L point mutants two resonances disappeared for each mutant. In both cases, the additional resonance disappearing had been assigned using methyl walks and PCSs (see also Fig. S10 for assignment of A446 and A448), i.e., in the case of A446G the second peak disappearing belongs to A448, and in the case of V352L the second peak is allocated to V391. Most likely, these mutations are associated with altered protein dynamics leading to exchange broadening and thus disappearance of the respective resonances. Therefore, the remaining disappearing resonances were assigned to A446 and V352. **(j)** For I358L two resonances disappeared upon mutation. I358 is in the loop that changes its orientation depending on whether P361 is *cis* or *trans*. Therefore, both resonances were assigned to I358, reflecting the presence of a P361 *cis-trans* mixture (compare Fig. 2d, showing two peaks disappearing upon mutation of A365 in the same loop). **(k)** I405 is located in a loop adjacent to the one containing A365 and had already been assigned using the methyl walk. The corresponding cross peak consists of two peaks, both disappearing in the I405L mutant and thus indicating that this loop is also affected by the P361 *cis-trans* isomerism. All peaks labeled with stars had been assigned using a methyl walk or PCSs. Red arrows assist backtracking signals showing CSPs upon mutation. **(b)** The concentration of LV-labeled wild-type P-domain was 4 µM. The concentration of all other wild-type P-domain samples was 500 µM (cf. Fig. S2). P-domain concentrations of mutants were: 9 µM LV‑labeled V387L **(b)**; a mixture of 115 µM each of A-labeled A442G, LV-labelled V304L, and I-labelled I405L **(e,f,k)**; a mixture of 60 µM each of LV-labeled V352L and A-labeled A446G **(h,i)**; a mixture of 75 µM each of A-labeled A444G and I-labeled I358L **(c,j)**; a mixture of 67 µM each of LV-labelled V378L and A-labeled A381G **(d,g)**. All spectra were acquired at 298 K on a Bruker Avance III HD 600 MHz NMR spectrometer equipped with a cryogenic probe.


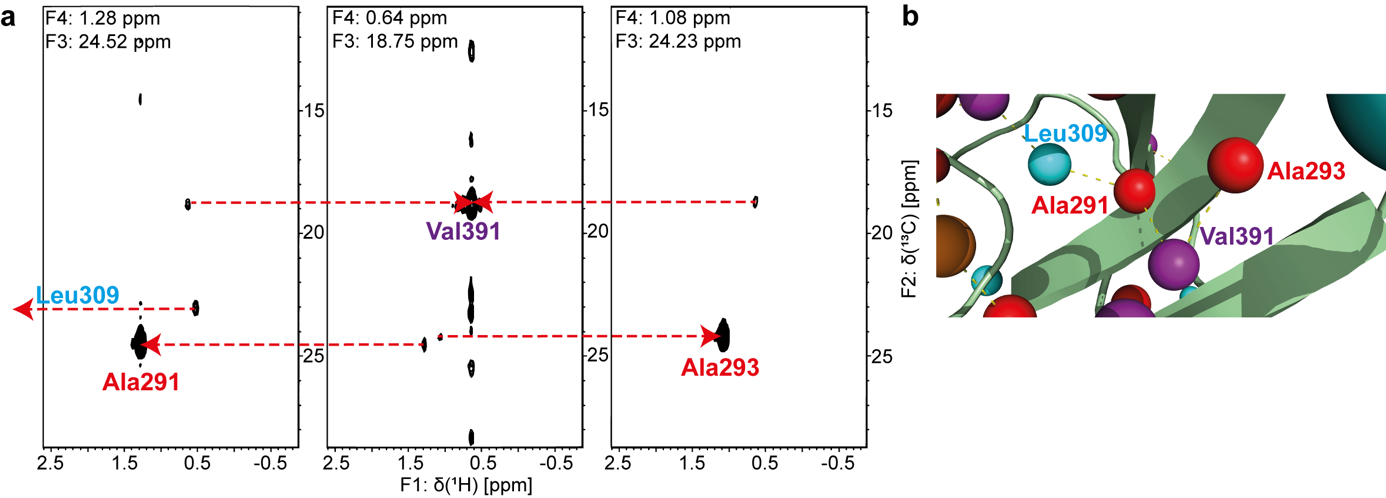


### Fig. S4: Assignment of Val391 using methyl-methyl NOEs.

Unambiguous assignment of Val391 exclusively based on methyl-methyl NOEs was essential for assigning V352, where two peaks disappeared upon V352L single point mutation, one of the peaks allocated to the methyl group of V352 and the other peak belonging to the Val391 methyl group (cf. Fig. S3 i). The cut-off distance was set at 5 Å. The methyl walk procedure is explained in the legend to Fig. S2.


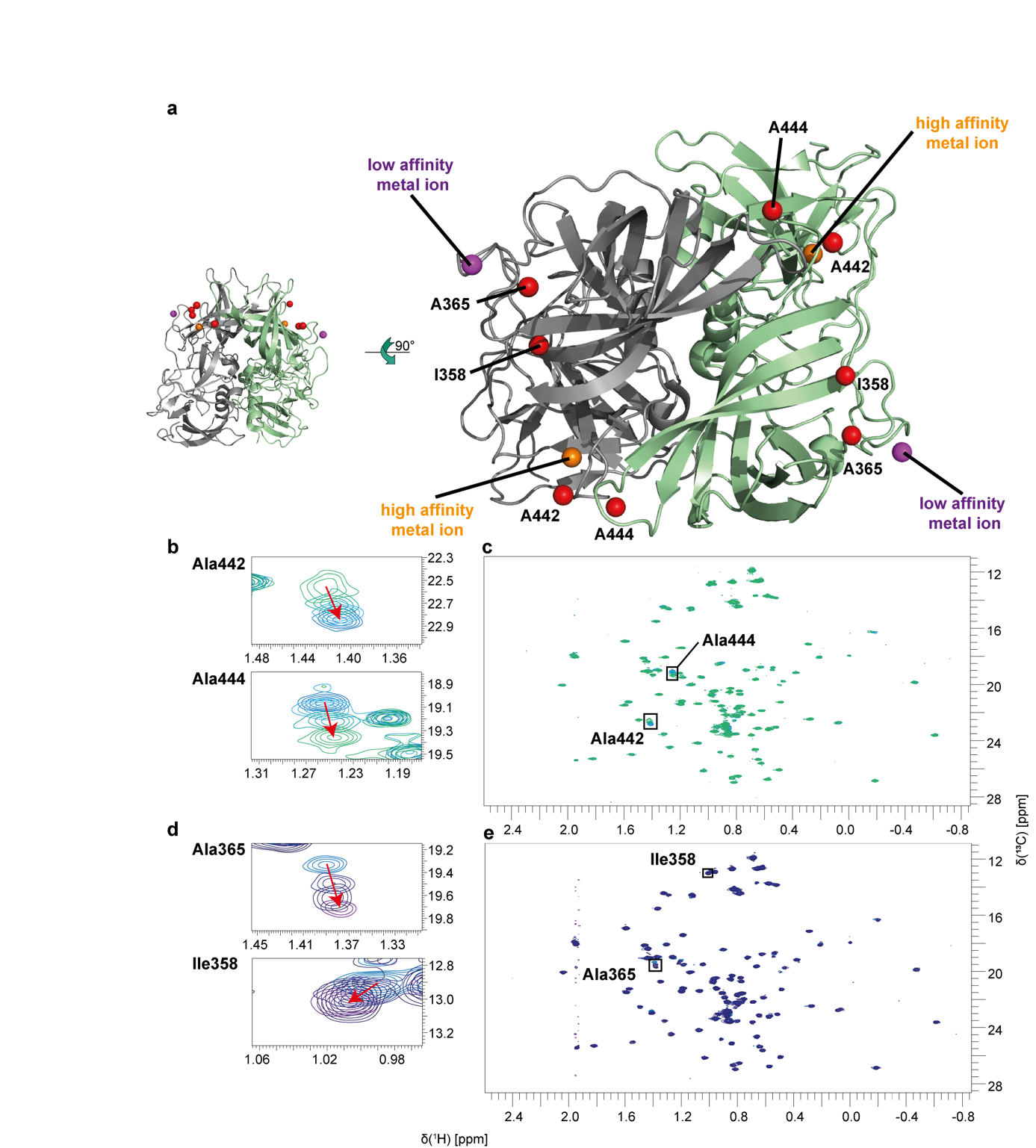


### Fig. S5: CaCl_2_ titration identifies two Ca^2+^ binding sites per monomer.

Titration of a P-dimer sample with CaCl_2_ exposes two distinct metal ion binding sites. **(b,c)** One set of peaks is characterized by CSPs going into saturation at about 550 µM CaCl_2_ (green - blue, 0 - 550 µM CaCl_2_). **(e,d)** A second set of peaks goes into saturation at about 20 mM CaCl_2_ (blue - magenta, 0.55 - 20 mM CaCl_2_). A comparison with crystal structure data **(a)** (Nelson et al. 2018) reveals that Ala444 and Ala442 **(b,c)** belong to the first, higher-affinity binding site. Ala365 and Ile358 **(e,d)** belong to the lower-affinity site. **(a)** Location of the methyl groups in the crystal structure relative to the position of the two metal ions. Spectra were acquired with 38 µM MILVA-labeled P-domain at 298 K on a 600 MHz spectrometer with cryo probe. For assignment of resonances see Figs. 2, S3, and S6.


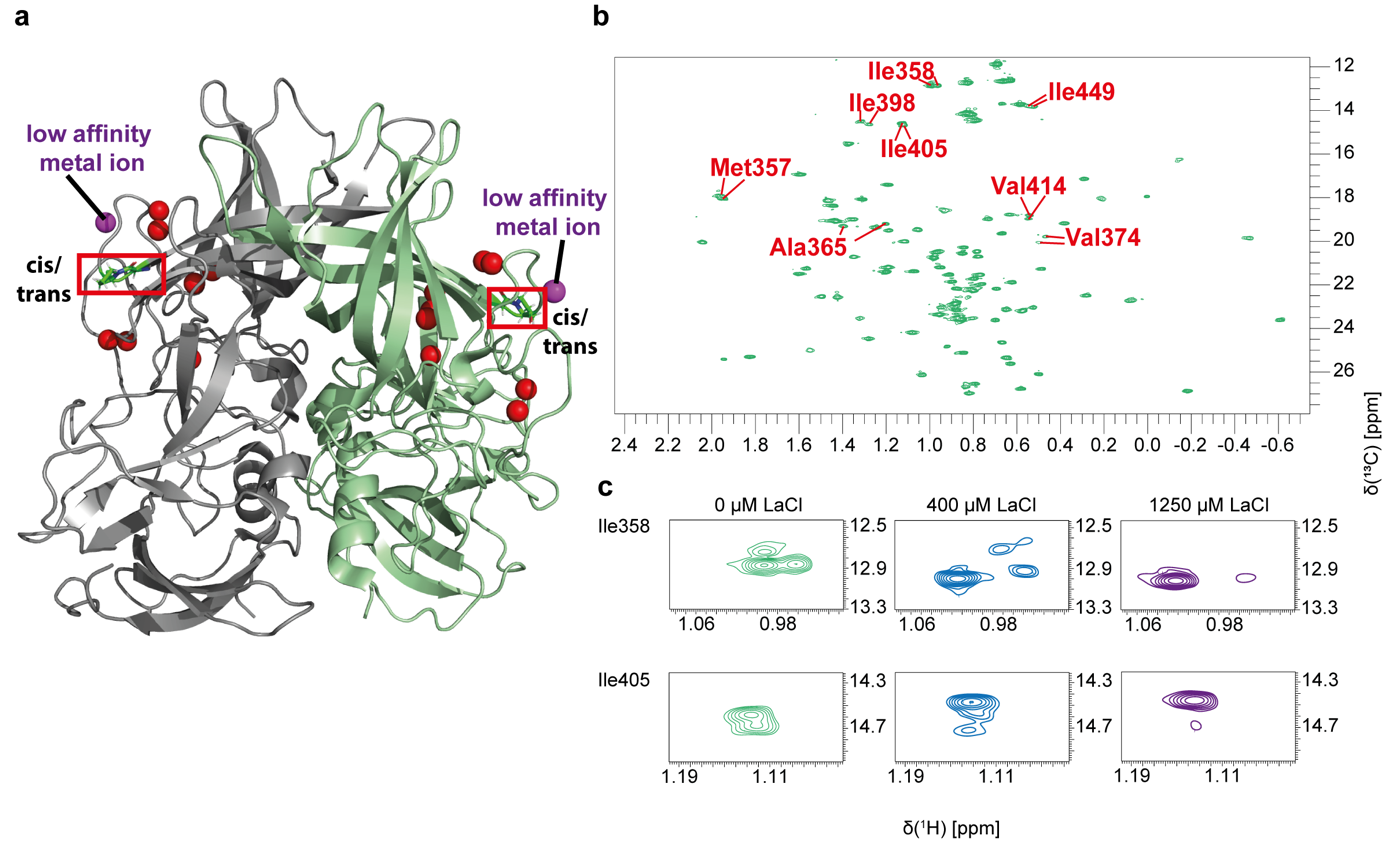


### Fig. S6: Titration with La^3+^ discloses two distinct forms of the MNV P-domain.

Titration with LaCl_3_ results in different behaviour **(c)** of methyl groups that exhibit double cross peaks in methyl TROSY spectra (cf. Fig. S3) due to the presence of a mixture of *cis-trans* isomers of P361. **(a)** Methyl groups giving rising to double cross peaks mapped on to the crystal structure (pdb 6e47) (Nelson et al. 2018). **(b)** Methyl TROSY spectrum of MNV P-dimers with resonances showing up as double cross peaks labeled. P-domain concentration was 38 µM (b+c) and spectra were acquired at 298 K on 600 MHz spectrometer with cryo probe.


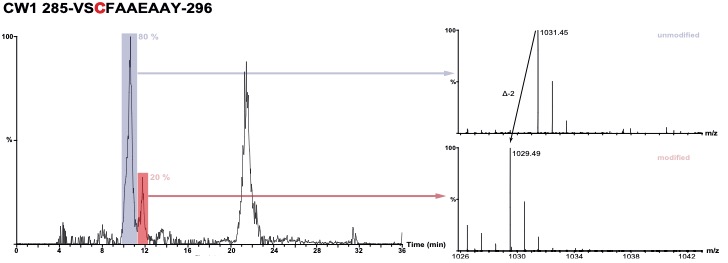


### Fig. S7: MS-based peptide mapping of 285-VSCFAAEAAY-296 of CW1 P Dimer

MS-based peptide mapping was performed to investigate potential modifications in the CW1 P dimer. Mapping performed after a pepsin digest showed good coverage for most of the peptide except for D403-G411 and V286-C288. To investigate these regions, further digests with chymotrypsin and Asp N were performed. Subsequent mapping produced good coverage for the residues D403-G411 and concluded that none were modified. The coverage for the second region was still inconclusive. Therefore, a combined Asp N/ chymotrypsin digest was performed. Chromatogram of this run can be seen **left**, with the corresponding peaks and ratio as indicated. MS analysis on unmodified on the **upper right**, modified on **lower right**. Investigation of the resulting peptides yielded a potential modification of C288, as indicated by a loss of ~2 Da. This mass shift may point to the formation of a sulfenylamide. Due to unclear fragment annotation during the analysis, the modification cannot be assigned to the peptide in question unambiguously. Unmodified (blue) and modified (red) C288 was found in an 80/20 ratio respectively. Digests were performed using 100 pmol protein. Resulting peptides were observed in MS using a Fusion Orbitrap Tribrid. Fragment annotation was achieved using MaxQuant (see supplemental information for MS).


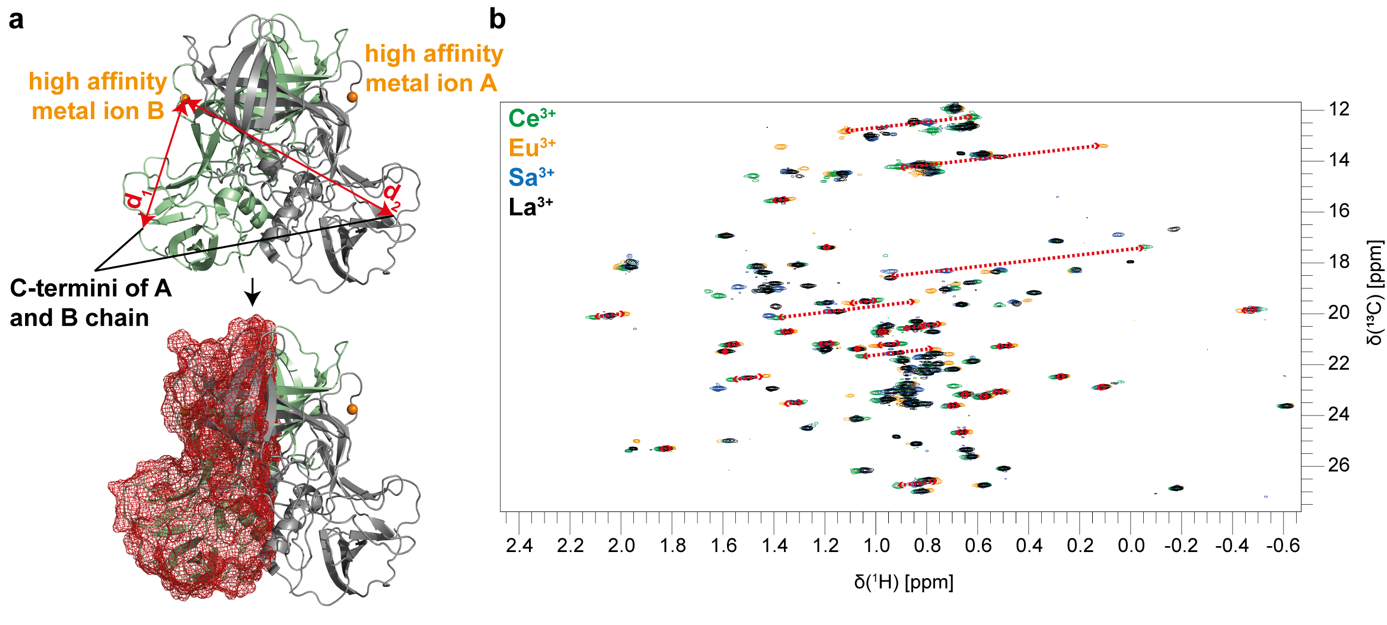


### Fig. S8: Preparing a crystal structure model for tensor fitting with Paramagpy.

**(a) Upper panel:** crystal structure model of MNV P-dimers (pdb 6e47) with bivalent metal ions shown as balls in the symmetric monomeric units, chains A (grey) and B (pale green). Only the higher affinity metal ion binding sites with a dissociation constant for Ca^2+^ ions of 138 μM (Creutznacher et al. 2021) are shown. It is assumed that only these two binding sites are fully occupied by lanthanide ions at the metal ion concentrations applied (400 µM LaCl_3_, 400 µM SaCl_3_, 450 µM EuCl_3_, and 400 µM CeCl_3_). At these concentrations, occupation of the low affinity metal ion binding sites is not significant and can be neglected (cf. Fig. S5). **Lower panel:** the red mesh covers those methyl groups that were included for fitting the alignment tensor to a single paramagnetic center, here site B, using the program Paramagpy (Orton et al. 2020). For the fitting process only the methyl group with the shorter distance to the given paramagnetic center, either located in chain A or in chain B is considered. To build this set of methyl groups for fitting, the distances d_1_ and d_2_ (red arrows) connecting the metal ion to symmetrically positioned atoms in chains A (gray) and B (green) are compared, and only the methyl group with the shorter distance is kept. **(b)** Methyl TROSY spectra of MNV P‑domain in the presence of saturating amounts (tenfold molar excess) of GCDCA and lanthanide ions. PCSs are exemplarily highlighted by dashed lines. The samples contained 31 µM MILVA-labelled P-domain, except for the sample containing Eu^3+^ where the concentration was 38 µM. Spectra were acquired on a Bruker 600 MHz Avance III HD spectrometer equipped with a cryogenic probe.


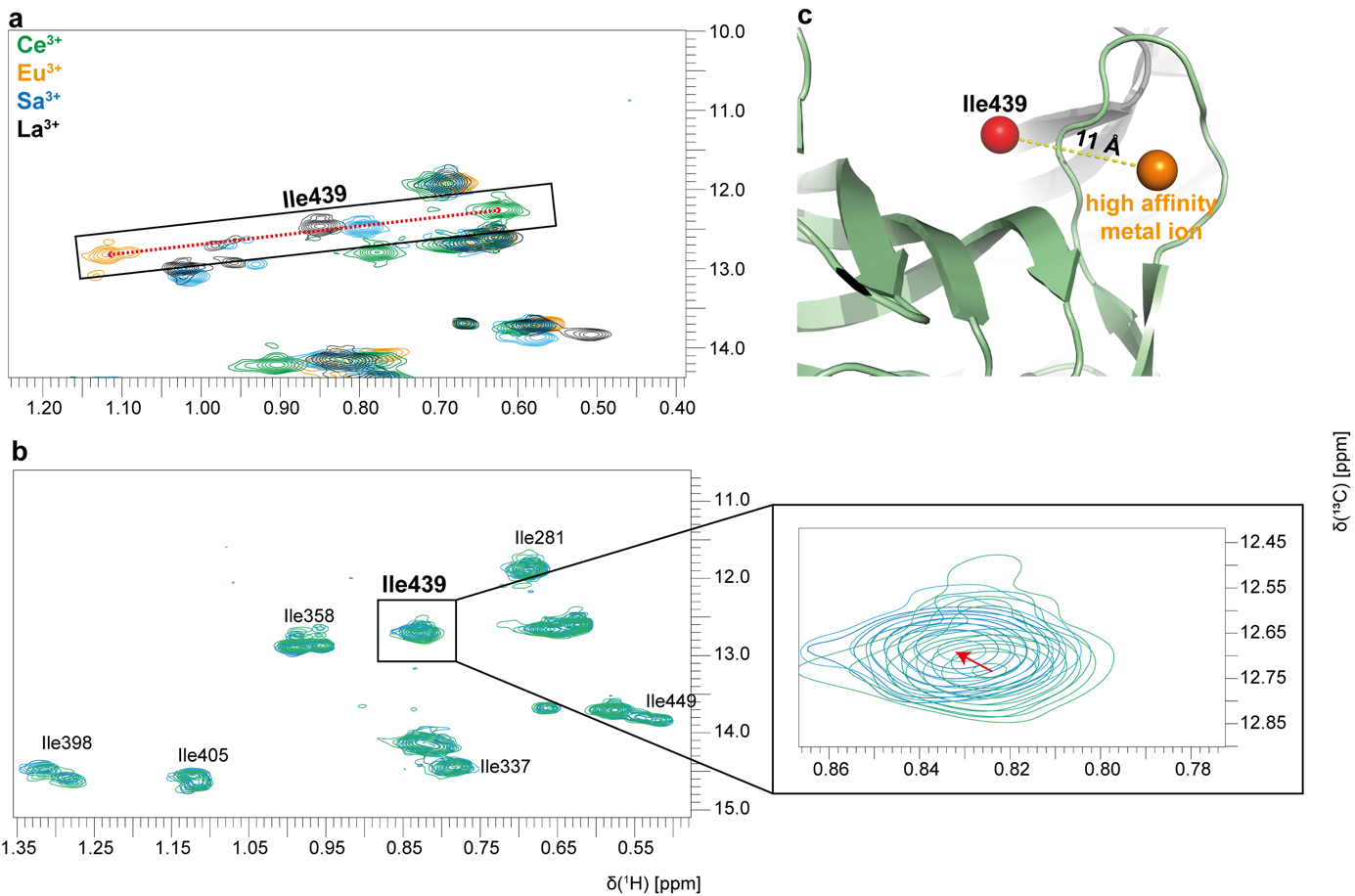


### Fig. S9: Assignment of Ile439.

**(a)** Section of a methyl TROSY spectrum of MILVA-labeled MNV P-domain. The box encompasses PCSs of an unassigned Ile residue in the presence of 450 µM EuCl_3_ (yellow), 400 µM SaCl_3_ (blue), or 400 µM CeCl_3_ (green). 400 µM LaCl_3_ was used as diamagnetic reference (black). **(b)** The unassigned Ile signal also shifts upon titration with CaCl_2_ (cf. Fig. S5) and belongs to the higher affinity metal ion binding site (cf. Fig. S5). **(c)** Within a sphere with a radius of 20 Å around the metal ion (orange) occupying the higher affinity site there is only one Ile residue that has not yet been assigned, Ile 439. Eperimental conditions are as given in the legend to Fig. S8 and S5.


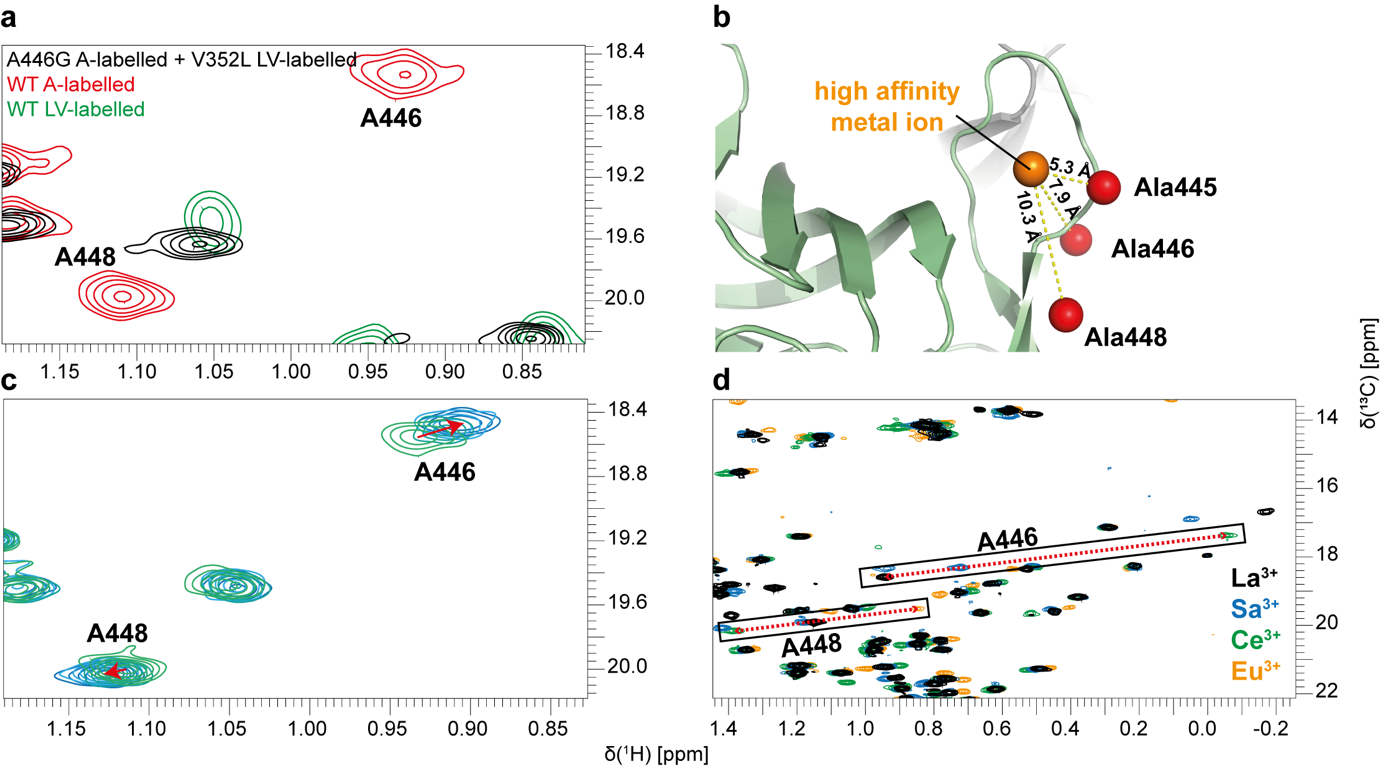


### Fig. S10: Assignment of Ala446 and Ala448.

**(a)** Methyl TROSY spectra of A-labelled mutant A446G and of LV‑labelled mutant V352L overlaid with the corresponding A- and LV-labeled spectra of wildtype P‑domains. For the conclusions here, only the A-labeled spectra are relevant. The LV-labeled sample has been measured together with the A-labeled sample to save measurement time. Upon the A446G mutation two resonances disappear, preventing an unambiguous assignment. Both peaks belong to the lower affinity metal ion binding site shown in the crystal structure model in **(b)**. **(c)** Shows the shifts of the two peaks upon titration with CaCl_2_ (cf. Fig. S5). **(d)** Comparison of experimental and theoretical PCSs allows to discriminate the two peaks, leading to the assignment shown. Calculated PCSs induced by Ce^3+^ (second round of refinement, cf. Tab. 1) for Ala446, Ala448 and Ala445 are 0.776, -0.237 and 1.651, respectively. The two resonances disappearing due to mutation show PCSs of -0.237 and 0.997, allocating them to Ala446 and Ala448. Sample conditions in **(a)** were 4 µM LV-labelled wildtype P-domain, 7 µM A-labelled wildtype P-domain and a mixture of 60 µM A-labelled A446G and LV-labelled V352L P-domain. For the experimental conditions in **(c)** and **(d)** see the legend of Fig. S5 and S8.


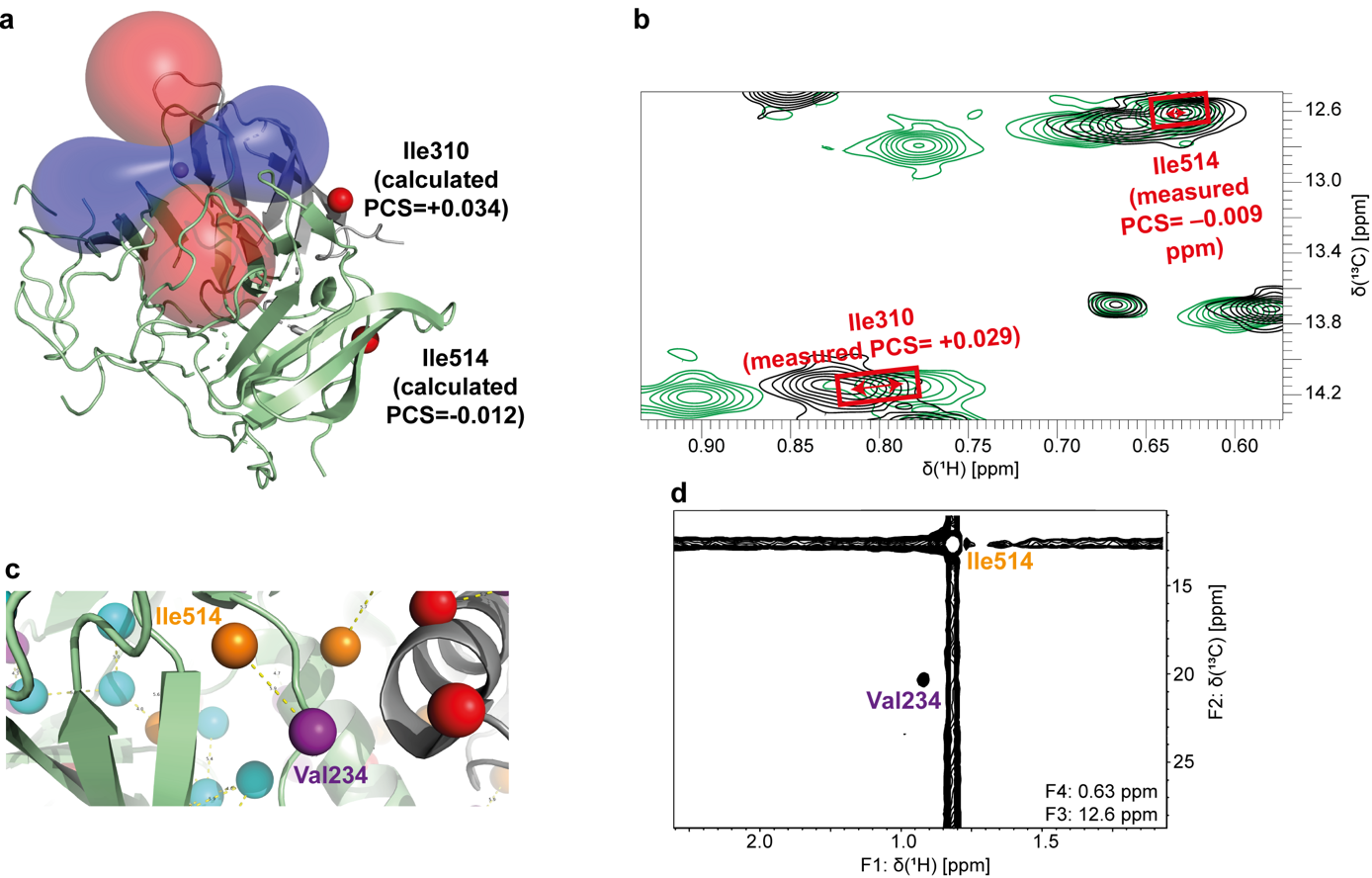


### Fig. S11: Assignment of Ile514, Ile310 and Val234.

**(a)** Isosurfaces of the alignment tensor in the presence of Ce^3+^ from the second round of refinement (See Fig. 3b and Tab. 1). Calculated (based on crystal structure pdb 6e47) PCSs are annotated with the unassigned Ile residues Ile310 and Ile514. **(b)** Comparison to experimental PCSs of two unassigned Ile peaks yields an unambiguous assignment of Ile310 and Ile514. Supporting evidence is found from methyl-methyl NOEs **(c, d)**. As methyl groups of Ile514 and Val234 are in close vicinity to each other **(c)**, an NOE is expected and indeed found experimentally in the corresponding F1-F2 plane of the 4D HMQC-NOESY-HMQC spectrum (cf. Fig. S2a) positioned at the methyl resonances of Ile514 in F3 and F4.


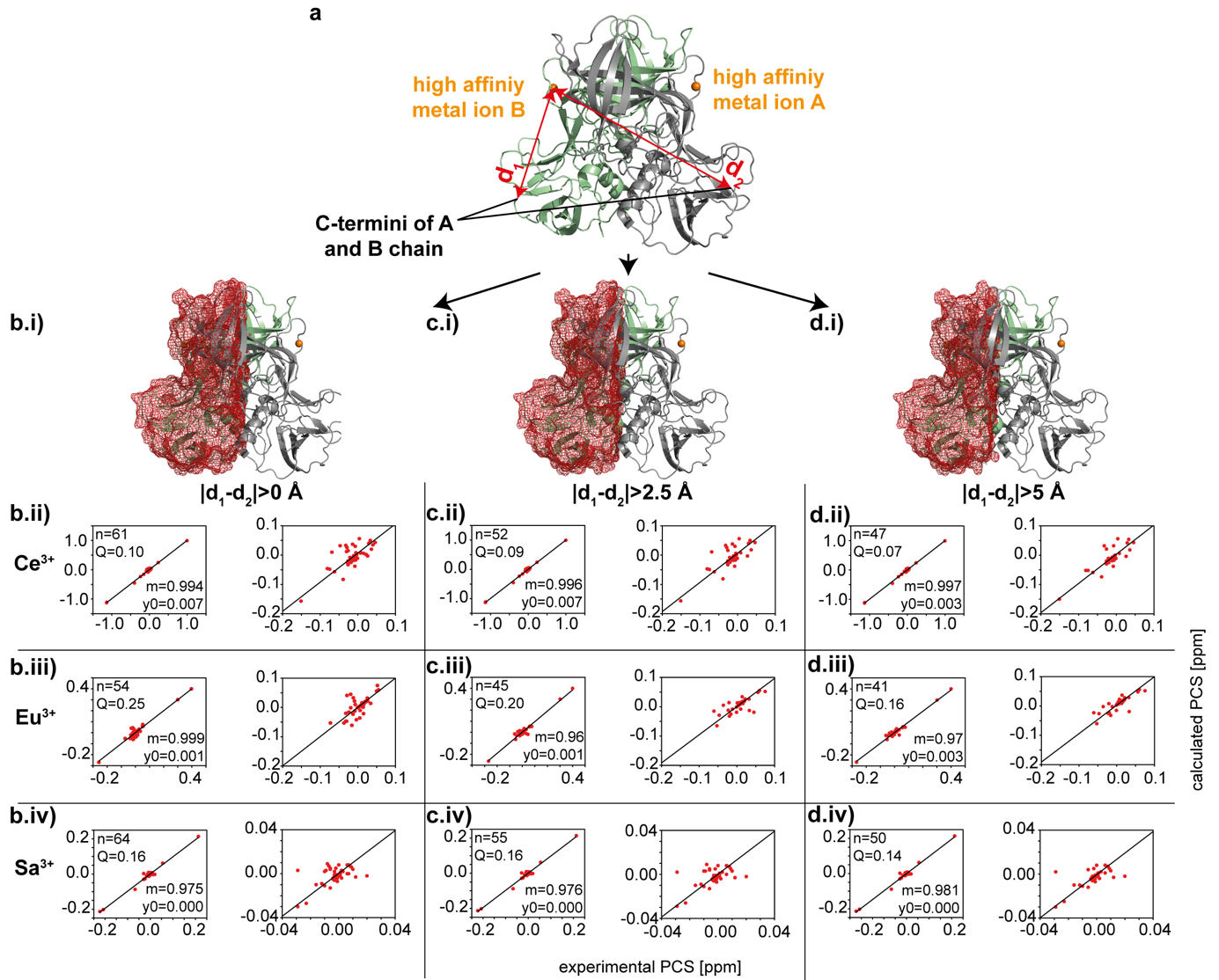


### Fig. S12: Validation of assignment using PCSs of Ce^3+^, Eu^3+^ and Sa^3+^.

The structural model including positions of metal ions ((a), pdb 6e47) was processed in order to fit alignment tensors with Paramagpy to a single paramagnetic centre. As the P-domain dimer has four metal ion binding sites we assumed that only the two higher affinity metal ion binding sites are significantly occupied at metal ion concentrations around 400 μM (see Fig. S5). We calculated distances from each atom from the A chain (grey) and the respective atom in the B chain (pale green) to the remaining metal ion binding to the B chain (metal ion B) and compared these distances (exemplary distances to metal ion B are shown in red for C-termini of A and B chain). For tensor fitting, we only considered atoms with the smaller of the two distances d1 and d2 and fulfilling the condition |d_1_-d_2_|>0 Å (covered by the red mesh in (b.i), same processing as in Fig. S8 a). The conditions for structure processing were altered to |d_1_-d_2_|>2.5 Å (atoms covered by red mesh shown in (c.i)) and to |d_1_-d_2_|>5 Å (atoms covered by red mesh shown in (d.i)). As coupling between the electron and the nucleus depends on r^-5^, the influence of the metal ion binding to the A chain (metal ion A in (a)) on the calculation of the alignment tensor is decreased due to structural processing conditions from (b.i) to (d.i). This is reflected in the Q-factors of the alignment tensors of Ce^3+^ (b.ii-d.ii), Eu^3+^ (b.iii-d.iii) and Sa^3+^ (b.iv-d.iv). n is the amount of assigned methyl group resonances for that PCSs were measured and therefore used for determination of the respective alignment tensor.

### Table S1: Primers used for site-directed mutagenis.

| **Mutant** | **Primer Sequence** |
| --- | --- |
| A365G | Forward: 5’-GACGACCAATGGAGATCAAGCGCC-3’  Reverse: 5’-GGCGCTTGATCTCCATTGGTCGTC-3’ |
| A381G | Forward: 5’-GTGTGACAGCCGGAGCCTCTC-3’  Reverse: 5’-GAGAGGCTCCGGCTGTCACAC-3’ |
| A442G | Forward: 5’-CAGATTGATACTGGAGATGCAGCGGCG-3’  Reverse: 5’-CGCCGCTGCATCTCCAGTATCAATCTG-3’ |
| A444G | Forward: 5’-GATACTGCGGATGGAGCGGCG-3’  Reverse: 5’-CGCCGCTCCATCCGCAGTATC-3’ |
| A446G | Forward: 5’-GCGGATGCAGCGGGAGAAGCCATC-3’  Reverse: 5’-GATGGCTTCTCCCGCTGCATCCGC-3’ |
| I358L | Forward: 5’-CTTCGAGATGCTATTGGGCCCGACG-3’  Reverse: 5’-CGTCGGGCCCAATAGCATCTCGAAG-3’ |
| V352L | Forward: 5’-GATAAGCTGAAACTAACGACCTTCGAGATG-3’  Reverse: 5’-CATCTCGAAGGTCGTTAGTTTCAGCTTATC-3’ |
| I405L | Forward: 5’-GGTTTCAGGACACCCTACCCGAATACAAC-3’  Reverse: 5’-GTTGTATTCGGGTAGGGTGTCCTGAAACC-3’ |
| V304L | Forward: 5’-GAACGGGTGAACTAGCGACCTTTACCCTG-3’  Reverse: 5’-CAGGGTAAAGGTCGCTAGTTCACCCGTTC-3’ |
| V378L | Forward: 5’-GTGTTTGCCAGTCTAACAGCCGCAGCC-3’  Reverse: 5’-GGCTGCGGCTGTTAGACTGGCAAACAC-3’ |
| V387L | Forward: 5’-CTCTGGATCTGCTAGATGGCCG-3’  Reverse: 5’-CGGCCATCTAGCAGATCCAGAG-3’ |

### Table S2: Final concentrations of precursors for MILVA-labeling of MNV-P-domains.

| **Amino acid** | **Precursor** | **Concentration** |
| --- | --- | --- |
| L^ProS^ , V^ProS^ | 2-^13^C-methyl-4-d_3_-acetolactate | 195 mg/L |
| I | 2-ketobutyricacid-4-^13^C-3, 3-d_2_ | 72 mg/L |
| A | L-alanine-^13^C-d_2_  succinate-d_4_ | 0.6 g/L  3.75 g/L |
| M | L-methionine-(methyl-^13^C) | 130 mg/L |

### Table S3: ^1^H, ^13^C HMQC acquisition parameters.

| **P-domain proteins** | **Spectra shown in figure** | **Data points in direct and indirect dimension** | **Number of scans** |
| --- | --- | --- | --- |
| A-labelled | S1a, S10a | 1024x256 | 80 |
| V-labelled | S1b | 1024x128 | 720 |
| LV-labelled | S1b, S3b. S10a | 1024x128 | 720 |
| MI-labelled | S1c | 1024x256 | 4 |
| ILV-labelled | S1c | 1024x256 | 4 |
| MILVA-labelled | 1b, 2d, S2c, S3 c-k | 512x512 | 4 |
| MILVA-labelled | S5b+c+d+e, S9b, S10c | 512x512 | 4 |
| LV-labelled V387L | S3b | 512x256 | 120 |
| A-labelled A365G | 2d | 512x256 | 8 |
| Mixture of A-labelled A442G, LV-labelled V304L and I-labelled I405L | S3e+f+k | 512x256 | 8 |
| Mixture of LV-labelled V352L and A-labelled A446G | S3h+I, S10a | 512x256 | 32 |
| Mixture of A444G and I-labelled I358L | S3c+j | 512x256 | 8 |
| LV-labelled V378L and A-labelled A381G | S4fd+g | 512x256 | 8 |
| MILVA-labelled | S6b+c | 512x256 | 8 for spectra with 0 and 400 uM LaCl_3_ and 4 for spectrum with 1250 uM LaCl_3_ |
| MILVA-labelled in presence of EuCl_3_ | S8b, S9a, S10d | 512x256 | 4 |
| MILVA-labelled in presence of SaCl_3_, LaCl_3_ or CeCl_3_ | S8b, S9a, S10d, S11b | 512x256 | 8 |

### Table S4: Parameters of alignment tensors of Ce^3+^, Eu^3+^ and Sa^3+^ using different structural processing approaches (see Fig. S10).

| Metal ion | | Ce^3+^ | | | Eu^3+^ | | | Sa^3+^ | | | Mg^2+^  (pdb 6e47) |
| --- | --- | --- | --- | --- | --- | --- | --- | --- | --- | --- | --- |
| Processing conditions | | \|d_1_-d_2_\|>0 Å  (S4 (b.i)) | \|d_1_-d_2_\|>2.5 Å  (S4 (c.i)) | \|d_1_-d_2_\|>5 Å  (S4 (d.i)) | \|d_1_-d_2_\|>0 Å  (S4 (b.i)) | \|d_1_-d_2_\|>2.5 Å  (S4 (c.i)) | \|d_1_-d_2_\|>5 Å  (S4 (d.i)) | \|d_1_-d_2_\|>0 Å  (S4 (b.i)) | \|d_1_-d_2_\|>2.5 Å  (S4 (c.i)) | \|d_1_-d_2_\|>5 Å  (S4 (d.i)) | - |
| ΔΧ_ax_ in  10^-32^ m^3^ | | -1.60 ±0.05 | -1.60 ±0.03 | -1.58±0.03 | 1.17±0.05 | 1.19±0.05 | 1.29±0.10 | -0.29±0.03 | -0.29±0.04 | -0.29±0.05 | - |
| ΔΧ_rh_ in  10^-32^ m^3^ | | -0.74 ±0.04 | -0.76 ±0.03 | -0.83±0.04 | 0.66±0.14 | 0.68±0.14 | 0.67±0.11 | -0.15±0.02 | -0.16±0.03 | -0.16±0.03 | - |
| Coordinates of origin in Angstroem | X | -16.3 ±0.5 | -16.4 ±0.6 | -16.6 ±0.5 | -15.6 ±0.7 | -15.6 ±0.8 | -16.7 ±1.2 | -16.5 ±0.5 | -16.5 ±0.5 | -16.5 ±0.4 | -14.9 |
|  | Y | 2.9 ±0.4 | 2.9 ±0.4 | 2.8 ±0.3 | 3.8 ±1.6 | 3.8 ±1.6 | 3.2 ±1.6 | 2.9 ±0.5 | 2.9 ±0.5 | 2.9 ±0.5 | 2.0 |
|  | z | -45.3 ±0.7 | -45.2 ±0.7 | -45.1 ±0.6 | -44.6 ±1.1 | -44.7 ±1.1 | -45.2 ±1.1 | -45.0 ±1.2 | -45.1 ±1.2 | -45.0 ±1.1 | -46.3 |
| Orinentation of principal axis of tensor in ° | α | 109.6 ±0.3 | 109.3 ±0.4 | 108.0 ±0.3 | 107.6 ±1.23 | 107.5 ±1.2 | 100.0 ±3.0 | 109.9 ±5.0 | 109.8 ±6.4 | 109.3 ±6.9 | - |
|  | β | 66.8 ±2.4 | 67.4 ±1.8 | 70.5 ±2.2 | 79.1 ±5.6 | 77.4 ±5.8 | 72.7 ±3.8 | 71.4 ±3.7 | 71.6 ±3.6 | 72.8 ±3.5 | - |
|  | γ | 155.9 ±2.0 | 156.4 ±1.9 | 157.7 ±2.0 | 169.8 ±47.8 | 169.7 ±42.6 | 170.7 ±22.1 | 164.3 ±10.2 | 164.9 ±17.6 | 165.4 ±12.9 | - |
| Amount of methyls included | | 61 | 52 | 47 | 54 | 45 | 41 | 64 | 55 | 50 | - |
| Q-factor | | 0.10 | 0.09 | 0.07 | 0.25 | 0.20 | 0.16 | 0.16 | 0.16 | 0.14 | - |

### Supplemental information for mass spectrometry-based peptide mapping of the MNV P‑domain

**Peptide mapping and PTM identification**

MNV CW1 P dimers were mixed with Chymotrypsin and Asp N proteases (Promega, USA) at a 1:30 ratio (protease:protein) and incubated for 2 h at 37°C in digestion buffer (100 mM Tris-HCl, 5 mM zinc acetate, 10 mM calcium chloride, pH 8) while shaking at 450 rpm. The mixture was then diluted with digestion buffer, aliquoted to 100 pmol MNV CW1 P dimer and frozen in liquid nitrogen.

Samples were thawed, centrifuged and injected onto a HPLC System (Agilent Infinity 1260, Agilent Technologies). Peptides were trapped (75 µL/min with 0.4 % formic acid in water, OPTI-TRAP cartridge for peptides, Optimize Technologies) and separated on a reversed-phase analytical column (PLRP-S for Biomolecules, Agilent Technologies) using a 27 min gradient of 8-40 % solvent B (solvent A: 0.4 % formic acid in water, solvent B: 0.4 % formic acid in acetonitrile) at 150 µL/min. Mass spectrometry was performed using an Orbitrap Fusion Tribrid in positive data-dependent MS/MS acquisition mode (Orbitrap resolution 120000, 1 microscan, HCD 30 with dynamic exclusion).

Precursor and fragment ions were searched and matched against a local protein database just containing the protein of interest in MaxQuant (version 1.6.5.0) using the Andromeda search engine (Tyanova et al. 2016) . Chymotrypsin and Asp N were selected as proteases and deamidation, oxidation, sulfation, hydroxyproline and disulfide bond formation were included as variable modifications with a maximum number of 5 modifications per peptide. Peptides between 4 and 30 amino acids length were accepted. The MaxQuant default mass tolerances for precursor (4.5 ppm) and fragment (20 ppm) ions defined for the Thermo Orbitrap instrument were used for data search. The minimum score for successful identifications was set to 0 for unmodified and 40 for modified peptides. For peptides carrying aPTM, spectra were checked manually and chromatographic peak areas where calculated in Xcalibur (Thermo Scientific) to obtain an unmodified/modified peptide ratio.

### References

References

Creutznacher R, Maass T, Ogrissek P, Wallmann G, Feldmann C, Peters H, Lingemann M, Taube S, Peters T, Mallagaray A (2021) NMR Experiments Shed New Light on Glycan Recognition by Human and Murine Norovirus Capsid Proteins. Viruses 13 (3). doi:10.3390/v13030416

Nelson CA, Wilen CB, Dai YN, Orchard RC, Kim AS, Stegeman RA, Hsieh LL, Smith TJ, Virgin HW, Fremont DH (2018) Structural basis for murine norovirus engagement of bile acids and the CD300lf receptor. Proc Natl Acad Sci U S A. doi:10.1073/pnas.1805797115

Orton HW, Huber T, Otting G (2020) Paramagpy: software for fitting magnetic susceptibility tensors using paramagnetic effects measured in NMR spectra. Magnetic Resonance 1 (1):1-12. doi:10.5194/mr-1-1-2020

Tyanova S, Temu T, Cox J (2016) The MaxQuant computational platform for mass spectrometry-based shotgun proteomics. Nat Protoc 11 (12):2301-2319. doi:10.1038/nprot.2016.136
